# Potato miR828 Is Associated With Purple Tuber Skin and Flesh Color

**DOI:** 10.3389/fpls.2018.01742

**Published:** 2018-12-11

**Authors:** Nicola Bonar, Michele Liney, Runxuan Zhang, Ceri Austin, Jimmy Dessoly, Diane Davidson, Jennifer Stephens, Gordon McDougall, Mark Taylor, Glenn J. Bryan, Csaba Hornyik

**Affiliations:** ^1^Cell and Molecular Sciences, The James Hutton Institute, Dundee, United Kingdom; ^2^Information and Computational Sciences, The James Hutton Institute, Dundee, United Kingdom; ^3^Environmental and Biochemical Sciences, The James Hutton Institute, Dundee, United Kingdom

**Keywords:** potato, *Solanum tuberosum*, micro RNA, miRNA, miR828, tasiRNA, anthocyanin, tuber

## Abstract

Anthocyanins are plant pigments responsible for the colors of many flowers, fruits and storage organs and have roles in abiotic and biotic stress resistance. Anthocyanins and polyphenols are bioactive compounds in plants including potato (*Solanum tuberosum* L.) which is the most important non-cereal crop in the world, cultivated for its tubers rich in starch and nutrients. The genetic regulation of the flavonoid biosynthetic pathway is relatively well known leading to the formation of anthocyanins. However, our knowledge of post-transcriptional regulation of anthocyanin biosynthesis is limited. There is increasing evidence that micro RNAs (miRNAs) and other small RNAs can regulate the expression level of key factors in anthocyanin production. In this study we have found strong associations between the high levels of miR828, TAS4 D4(-) and purple/red color of tuber skin and flesh. This was confirmed not only in different cultivars but in pigmented and non-pigmented sectors of the same tuber. Phytochemical analyses verified the levels of anthocyanins and polyphenols in different tissues. We showed that miR828 is able to direct cleavage of the RNA originating from *Trans-acting siRNA gene 4* (*TAS4*) and initiate the production of phased small interfering RNAs (siRNAs) whose production depends on RNA-dependent RNA polymerase 6 (RDR6). MYB transcription factors were predicted as potential targets of miR828 and TAS4 D4(-) and their expression was characterized. *MYB12* and *R2R3-MYB* genes showed decreased expression levels in purple skin and flesh in contrast with high levels of small RNAs in the same tissues. Moreover, we confirmed that *R2R3-MYB* and *MYB-36284* are direct targets of the small RNAs. Overall, this study sheds light on the small RNA directed anthocyanin regulation in potato, which is an important member of the *Solanaceae* family.

## Introduction

Anthocyanins are natural plant pigments in many flowers, fruits and vegetables providing red, blue and purple hues attractive for flower pollinators and also abiotic and biotic stress resistance (Jaakola, 2013; Lotkowska et al., 2015; Liu et al., 2018). Model and crop plants such as *Arabidopsis*, maize, *Petunia* and *Antirrhinum* have been studied extensively for flavonoid biosynthesis and a number of transcription factors were revealed regulating structural genes of the biosynthetic pathway (Holton and Cornish, 1995; Saito et al., 2013). *Solanaceae* species (such as tomato, eggplant, pepper and potato) show large diversity in the color of fruits or tubers and accumulate anthocyanins at various levels. Tissue color is strongly influenced by the anthocyanin and carotenoid pathways and anthocyanins accumulate as water soluble vacuolar flavonoids (Dhar et al., 2015). Potato (*S. tuberosum* L.) accumulates varying levels of anthocyanins in tuber tissues which are consumed widely around the world. Potato is an important global food crop which is cultivated for tubers (underground storage stem), rich in starch, nutrients and vitamins. Its global production of ∼376 million tons in 2016 shows its importance and increasing role in food security^1^. The appearance of anthocyanins in tuber skin and flesh can strongly influence consumer choice (Jemison et al., 2008).

Potato anthocyanin accumulation was extensively studied in different cultivars previously (Brown et al., 2007; Oertel et al., 2017). Potato tubers are potent sources of antioxidants and some studies reported that tubers are rich in polyphenols (De Jong et al., 2004; Stushnoff et al., 2008; Lee et al., 2016). Additionally, these bioactive compounds have potential health benefits including anticancer and immunomodulatory activities (Zuber et al., 2015; Chandrasekara and Josheph Kumar, 2016). Anthocyanin accumulation is influenced by the balance between biosynthesis and degradation. A conserved network of activators and repressors regulating anthocyanin pigmentation was identified in eudicots highlighting the complexity of the process (Albert et al., 2014). Phenylalanine is the initial precursor of the flavonoids biosynthetic pathway and various enzymes catalyze the production of the intermediate and end products regulated by a common set of transcription factors including MYB, MYC, and WD40 family members (Dhar et al., 2015; Liu et al., 2018). Previously, it was demonstrated that in diploid potato three genetic loci control anthocyanin accumulation in tuber skin: *P*, *R*, and *I* (Dodds and Long, 1955). *P* is required for production of purple delphinidin-based anthocyanins, *R* for pelargonidin-based pigments and *I* plays a role in tissue specific expression in tuber skin. Later these loci have been mapped in potato; for locus *R* dihydroflavonol 4-reductase (*dfr*) and for locus *I* an R2R3 MYB transcription factor designated an2 were identified. Locus *P* was found to encode flavonoid 3′,5′-hydroxylase (De Jong et al., 2004; Jung et al., 2005; Zhang et al., 2009). R2R3 MYB transcription factors were further identified in potato and validated by transient assays influencing anthocyanin biosynthesis including *AN1*, *MYBA1*, and *MYB113* (Liu et al., 2016). Over-expression of MYB transcription factor gene *StMtf1* resulted in the activation of phenylpropanoid biosynthetic pathway and tubers contained increased levels of chlorogenic acid and anthocyanins (Rommens et al., 2008). Genome-wide expression analysis of transcripts was performed in order to identify key regulators of anthocyanin production and establish a regulatory network to better understand the regulatory mechanisms. Using the same tuber but differently pigmented sectors, 27 differentially expressed genes were identified between white and purple sectors including a single domain MYB transcription factor (Stushnoff et al., 2010). Sprouts, originating from tubers with contrasting pigmentation of three cultivars, were analyzed for metabolites and gene expression using RNA sequencing; anthocyanin and transcriptional changes were found among 22 compounds and 119 transcripts including anthocyanin biosynthesis genes, hormones, transcription factors and signaling related genes (Cho et al., 2016).

Anthocyanin content of plant tissues is influenced by short (21–24nt in length), non-coding, single stranded RNA molecules called micro RNAs (miRNAs) (Rock, 2013). miRNAs are established from the genome and are transcribed by RNA polymerase II (pri-miRNA) (Vaucheret, 2006; Borges and Martienssen, 2015). The transcript forms a strong secondary structure which is recognized and processed by an RNase III type endoribonuclease (DICER-LIKE 1, DCL1); the cleaved product has a stem-loop (hairpin) structure with characteristic free energy (pre-miRNA). The hairpin RNA is further cleaved by DCL1 and a double stranded intermediate molecule is established which will be methylated at the 3′ end by HUA ENHANCER 1 (HEN1) to prevent degradation (Yu et al., 2005). The miRNA incorporates into the effector complex (RNA Induced Silencing Complex, RISC) where it provides sequence specificity and directs the complex to the relevant RNA molecules. The other strand of the intermediate double stranded RNA (star sequence) is usually degraded. Overall, miRNAs act at the post-transcriptional level, silencing the expression of target genes through RNA cleavage or translational inhibition (Rogers and Chen, 2013).

Previous studies implied a role for several miRNAs in tissue color formation (Rock, 2013). In *Arabidopsis thaliana* stem, miR156 promotes accumulation of anthocyanins whereas decreased miR156 activity results in higher levels of flavonols which is under the regulation of miR156 targeted SQUAMOSA PROMOTER BINDING PROTEIN-LIKE (SPL) genes (Gou et al., 2011). In lychee, miR156a is differentially expressed during fruit ripening and can influence anthocyanin accumulation via the regulation of SPL1/2 (Liu et al., 2017). In tomato, miR858 was identified as a negative regulator of anthocyanin biosynthesis regulating the expression of MYB transcription factor genes (Jia et al., 2015). In *A. thaliana*, over-expression of miR828 resulted in reduced expression levels of MYB transcription factors and lower levels of anthocyanins (Yang et al., 2013). Moreover, miR828 and miR858 were shown to regulate arabidopsis trichome and cotton fiber development via *MYB2* gene function (Guan et al., 2014).

Some miRNAs, including miR828, are able to trigger the generation of secondary phased small interfering RNAs (phasiRNAs) (Zheng et al., 2015). After the cleavage of target transcript, the 3′ cleaved RNA product is converted into a double stranded RNA molecule by RNA-dependent RNA polymerase 6 (RDR6) which is processed by DICER-LIKE 4 (DCL4) enzyme into 21nt long siRNAs. Some of the phasiRNAs can regulate the expression of target genes and are called *trans*-acting siRNA (tasiRNAs) (Rajagopalan et al., 2006). miR828 targets a non-coding RNA named *Trans-acting siRNA gene 4* (*TAS4*) and initiates tasiRNA production. Some of the generated tasiRNAs accumulate to a high level in plants and can target mRNAs of other genes (e.g., MYB transcription factors) influencing important biological pathways (Rajagopalan et al., 2006). One of the tasiRNAs is TAS D4(-) [previously TAS4-siR81(-)] and it was shown that a MYB transcription factor (PAP2/MYB90) is its direct target in *A. thaliana* controlling expression of anthocyanin, flavonoid and phenylpropanoid biosynthetic genes (Borevitz et al., 2000; Rajagopalan et al., 2006; Luo et al., 2012). Additionally, TAS4 D4(-) can target *PAP1* and *MYB113* *MYB* genes in *A. thaliana* and its level is increased by exogenous treatment with sucrose and glucose (Luo et al., 2012). In parallel, exogenous sucrose increased the anthocyanin level in plants. Increased *TAS4* derived tasiRNA production was observed in phosphate (Pi) deficient shoots of *A. thaliana* and an auto-regulatory mechanism was uncovered involving *PAP1/MYB75*, miR828 and TAS4 D4(-) which regulate anthocyanin biosynthesis (Hsieh et al., 2009). In apple, miR858, miR828, and TAS4 D4(-) were identified in a small RNA high-throughput sequencing study and it was found that they target up to 81 *MYB* genes (Xia et al., 2012). Potato has been previously characterized for miRNAs (Zhang et al., 2013; Lakhotia et al., 2014) but our current knowledge about the function of miR828 in crop plants including *S. tuberosum* is limited.

The previously described studies show that the anthocyanin biosynthetic pathway and its genetic regulation are well understood but other levels of anthocyanin regulation, including post-transcriptional, remain to be fully elucidated. In this study, the role of miR828 was investigated in potato tuber color formation. We identified and characterized potato miR828 in different cultivars and showed that miR828 can trigger the generation of tasiRNAs. A strong association was found between the presence of miR828 and the purple color of tuber tissues. The anthocyanin and polyphenol content of the investigated cultivars confirmed that their accumulation will result in purple/red tissue formation. We predicted potential targets of miR828 and TAS4 D4(-), members of the MYB transcription factor family were investigated for their expression in potato tuber skin and flesh and they were identified as direct targets of the small RNAs.

## Results

### Anthocyanins Accumulate in a High Manner in Purple Tuber Tissues

**FIGURE 1:**
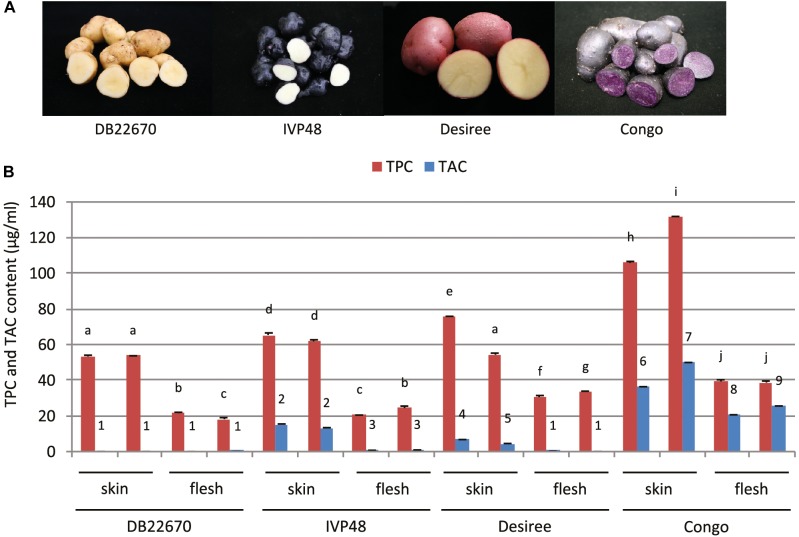
Potato cultivars used in this study and total polyphenol and anthocyanin content of tuber skin and flesh tissues. **(A)** Photos of intact and cut tubers of cultivars used in this study. **(B)** Total polyphenol and anthocyanin content (TPC and TAC, respectively) of tuber skin and flesh tissues. Error bars show standard errors (SE). Bars with different symbols are significantly different by pairwise Student’s *T*-test (*p* < 0.05). Different letters show significant differences between TPC values; different numbers represent significant differences between TAC values.

In our study, a number of potato cultivars were chosen for studying the role of miR828 having contrasting tuber skin and flesh colors (Figure 1A). DB22670 (*S. tuberosum* group *Phureja*) has white/yellow skin and white flesh tissues; IVP48 (*S. tuberosum* group *Phureja*) has dark purple/navy blue skin and white flesh; Desiree (*S. tuberosum* L. ‘Desiree’) is a well-known cultivar used widely in Europe with red skin and yellow flesh and Congo (*S. tuberosum* L. ‘Congo’) has deep blue/purple skin with the same color of tuber flesh.

Phytochemical analysis was carried out to provide more precise data about the compounds which can influence the color of tuber skin and flesh tissues. Tuber skin and flesh tissues were collected separately from the cultivars studied for small RNA expression. Samples were powdered under liquid nitrogen and freeze-dried followed by extraction, analysis of total anthocyanin and polyphenol content and analysis by liquid chromatography–mass spectrometry (LC-MS). Total polyphenol and anthocyanin contents (TPC and TAC, respectively) were determined (Figure 1B). TPC was higher in skin tissues compared to flesh samples in all cultivars. The highest level was detected in skin samples of Congo tubers whereas the skin tissues of IVP48 and Desiree had similar but lower levels and the lowest TPC was determined in DB22670 skin. The flesh samples had more similar levels of TPC with the highest in Congo then Desiree with IVP48 having similar lower values.

As expected, the total phenol content of the samples did not correlate with color but total anthocyanin content was more closely associated with visible tissue color. In cultivar DB22670 with yellow skin and white flesh, anthocyanins were barely detectable with only a very low amount in one flesh sample. IVP48 has dark blue skin and white flesh color and gave a higher level of TAC in skin compared to a low abundance in flesh, which might originate from residual unpeeled skin tissues. Desiree also had a higher TAC in skin than flesh tissues which reflects their red and yellow colors, respectively. The highest total anthocyanin content was measured in Congo skin (deep purple) and flesh (purple). LC-MS data (Supplementary Figure S1) confirmed the TAC data with the highest levels of anthocyanins found in the Congo skin and flesh. This analysis also demonstrated that the different red and purple tissue colors were influenced by their different anthocyanin profiles. For example, the purple cultivar Congo was enriched in malvanin, a coumaroylated malvidin anthocyanin well documented to accumulate in purple fleshed potatoes (Oertel et al., 2017), whereas the red skin coloration of Desiree was associated with the accumulation of pelanin and peonanin (Mori et al., 2010).

### miR828 Accumulates at High Levels in Purple Potato Tissues

**FIGURE 2:**
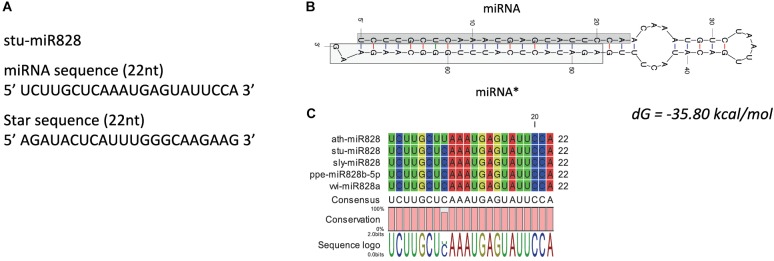
miR828 in potato. **(A)** stu-miR828 miRNA and star sequence. **(B)** The predicted secondary structure of stu-miR828 (Mfold) with free energy (*dG*); miRNA^∗^ shows the star sequence. **(C)** Alignment of arabidopsis, potato, tomato, peach and grapevine miR828.

High-throughput sequencing analysis was used to identify the miR828 sequence as this miRNA was not identified previously in potato [miRBase 22, (Kozomara and Griffiths-Jones, 2014)]. Only one family member of miR828 was found with 22 nt length in *S. tuberosum* L. ‘Desiree’ tuber tissues. The plant material for high-throughput sequencing originates from a heat stress study (Hancock et al., 2014). Figure 2A shows the predicted mature miRNA and star sequences of potato miR828 (stu-miR828) with the predicted secondary structure of the pre-miRNA forming a stem loop (hairpin-like) structure with free energy of -35.80 kcal/mol (Figure 2B). stu-miR828 shows identity to the previously described miR828 sequences from tomato, peach and grapevine but contains one mismatch compared to miR828 in *A. thaliana* (Figure 2C) (Kozomara and Griffiths-Jones, 2014; Singh et al., 2016). stu-miR828 fulfills the updated requirements (Axtell and Meyers, 2018) for being a miRNA because of the structure of the pre-miRNA and both the miRNA and the star sequence were found in the sequencing analysis; the details of predicted stu-miR828 can be found in Supplementary Table S1.

**FIGURE 3:**
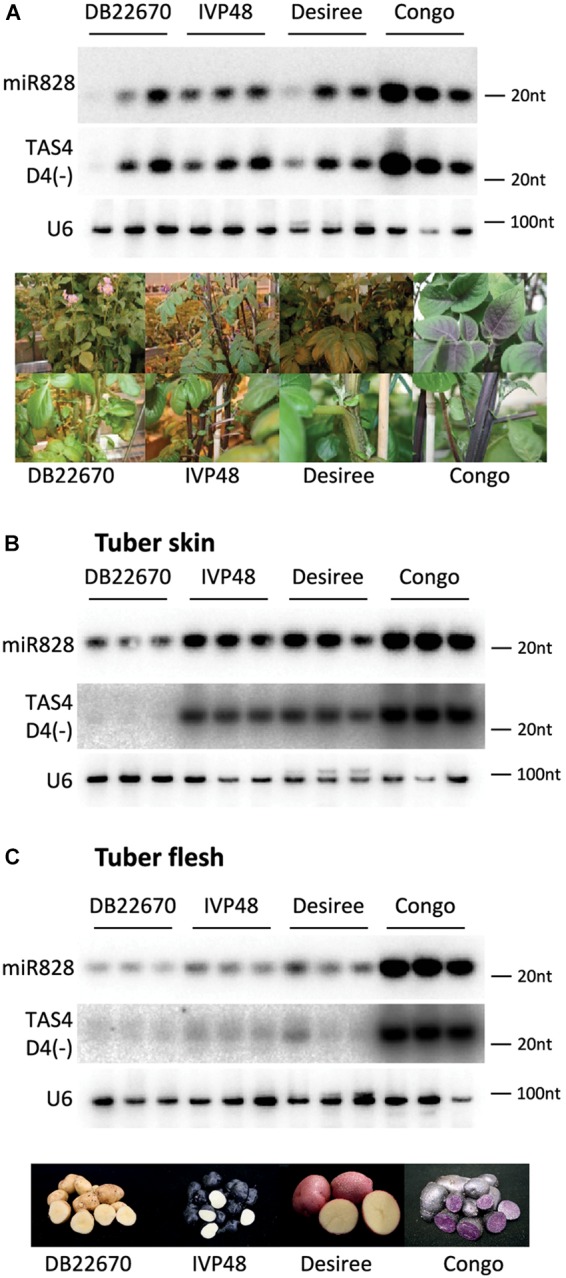
RNA gel blot analysis of small RNAs. Potato leaf **(A)**, tuber skin **(B)**, and flesh **(C)** tissues were used to detect small RNAs with radioactively labeled probes specific to miR828 and TAS4 D4(-). U6 was used as a loading control. Three biological replicates of plant tissues were used for small RNA detection indicated by solid line and the name of cultivars. Photos on **(A,C)** show leaves, stems, and tubers of cultivars.

RNA gel blot analysis was used to investigate the accumulation of miR828 in different tissues (Figure 3). In leaves, miR828 showed variable accumulation among the cultivars but higher miR828 level was found in Congo leaves which contain purple sectors in the leaflets (Figure 3A). However, miR828 accumulation showed strong association with the presence of purple color in potato tubers. Purple skin and flesh tissues accumulate miR828 in high levels compared to white/yellow tissues. In the purple/red/blue skin samples of IVP48, Desiree and Congo the accumulation level of miR828 is higher than in DB22670 which has white/yellow skin. Additionally, miR828 showed high level only in Congo tuber flesh tissue which is deep purple compared to other cultivars with white or yellow tuber flesh colors (Figures 3B,C). This result indicates an association between the presence of miR828 and purple/deep blue colors in tuber skin and flesh.

### miR828 Can Initiate Secondary Small RNA Production in Potato

**FIGURE 4:**
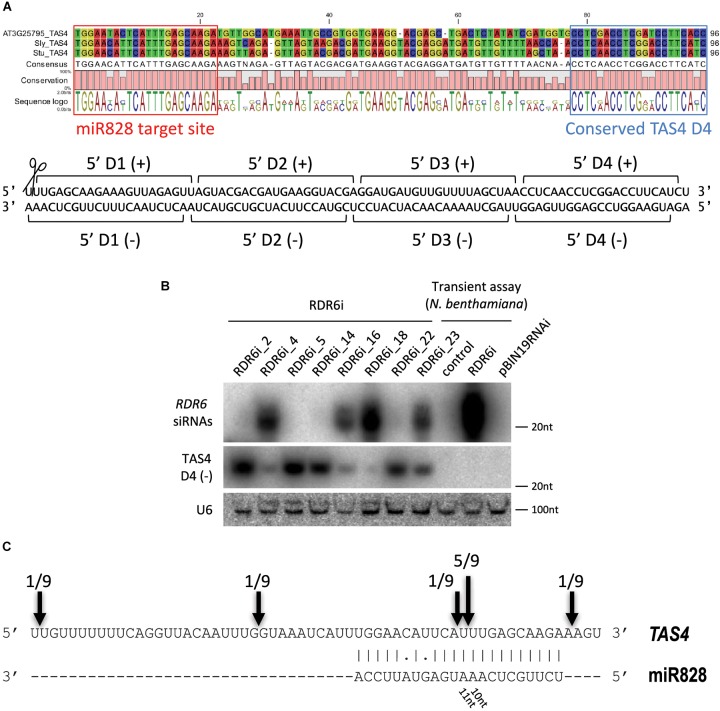
Analysis of potato *TAS4*. **(A)** Sequence alignment of *TAS4* sequences of *Arabidopsis thaliana*, tomato and potato; the miR828 target site and TAS4 D4(-) are marked. Below the alignment the double stranded (ds) DNA sequence shows the cut *TAS4* RNA with the first four phased siRNAs. **(B)** RNA gel blot analysis of RDR6i lines; *Nicotiana benthamiana* transient assay was used as a control (control: non-infiltrated leaf; RDR6i: leaf infiltrated with RDR6i construct; pBIN19RNAi: leaf infiltrated with empty vector). Radioactively labeled random probe of RDR6 amplicon and probe specific to TAS4 D4(-) were used to visualize RDR6i derived siRNAs and TAS4 D4(-). U6 was used as a loading control. **(C)** RLM 5′ RACE was used to identify free ends of *TAS4* RNA. Arrows and numbers indicate free RNA ends with the frequency of cut points identified; the predominant cut occurs between 10/11 nucleotides of miR828.

In previous studies it was found that miR828 can initiate secondary small RNA production targeting *TAS4* (Fei et al., 2013). In order to find *TAS4* orthologs in potato we searched the potato genome database for *A. thaliana TAS4* (AT3G25795) and the *TAS4* locus sequence from tomato [T. Dalmay, unpublished, (Singh et al., 2016)]. Using transcripts and non-redundant CDS database we could not identify the *TAS4* locus in potato. However, using the superscaffold database we identified a region of the genome which showed moderate similarity to the *TAS4* locus (Supplementary Figure S2). The *A. thaliana*, tomato and potato *TAS4* sequences were aligned, the miR828 target site was predicted using psRNATarget software (Dai and Zhao, 2011) and a conserved *TAS4* tasiRNA was identified (Figure 4A). TAS4 D4(-) tasiRNA is very abundant in other species and the sequence in potato shows 100% similarity to the corresponding tomato tasiRNA. Previously, this tasiRNA was named as TAS4-siR81(-) and it was shown that a MYB transcription factor (PAP2/MYB90) is its direct target in *A. thaliana* controlling expression of anthocyanin, flavonoid, and phenylpropanoid biosynthetic genes (Borevitz et al., 2000; Rajagopalan et al., 2006; Luo et al., 2012).

To investigate the expression level of TAS4 D4(-), small RNA gel blot analysis was performed using the samples which were analyzed for miR828 expression. TAS4 D4(-) was easily detectable by RNA gel blot analysis and its abundance was correlated with the level of miR828 (Figure 3). In leaf, TAS4 D4(-) showed variable accumulation among the cultivars having the highest accumulation in Congo leaves similarly to miR828 (Figure 3A). However, higher expression level was found in tuber skin of cultivars with purple or red color (IVP48, Desiree and Congo), especially in Congo skin samples; TAS4 D4(-) was hardly detectable in skin tissues of DB22670 (Figure 3B). A similar accumulation level of TAS4 D4(-) was found in tuber flesh samples compared to miR828 expression. High expression level was detected in Congo tuber flesh with purple color but in DB22670, IVP48 and Desiree flesh which are either white or yellow, TAS4 D4(-) accumulated at low levels (Figure 3C). This accumulation pattern strongly suggests that miR828 can trigger the generation of the tasiRNA and TAS4 D4(-) was present in purple tissues more abundantly.

Biogenesis of tasiRNAs requires RDR6 and DCL4 (Peragine et al., 2004; Vazquez et al., 2004; Xie et al., 2005). To test whether TAS4 D4(-) generation depends on RDR6 function in potato, transgenic Desiree plants were generated using an RNAi vector expressing a hairpin construct against RDR6 (PGSC0003DMT400060673). Various RDR6i transgenic lines were obtained with different efficiency for the silencing of endogenous RDR6 (Supplementary Figure S3). The most efficiently silenced lines showed about 50% residual expression of endogenous RDR6 gene compared to empty vector transformed potato lines. The efficiency of the hairpin construct was monitored through the generation of RDR6 specific small interfering RNAs (siRNAs). Some lines (RDR6i_4, 16, 18, and 23) showed reduction of RDR6 expression; in parallel a high abundance of RDR6 specific siRNAs was detected in these plants confirming that the silencing against RDR6 is effective (Figure 4B). Transient assay in *Nicotiana benthamiana* was used as a control to show the efficiency of the hairpin construct; we could detect RDR6 derived siRNA only in the case of the hairpin infiltration. Having altered expression of RDR6 in Desiree lines we checked whether TAS4 D4(-) expression depends on RDR6. Small RNA gel blot analysis was performed to visualize the accumulation of TAS4 D4(-). Higher amount of TAS4 D4(-) was detected in transgenic plants where the silencing against RDR6 was weak (RDR6i_2, 5, 14, and 22). In contrast, we detected lower expression level of TAS4 D4(-) in transgenic plants where the silencing of RDR6 occurred at a high level (RDR6i_4, 16, 18, and 23); these plants showed high levels of RDR6 siRNAs but lower levels of TAS4 D4(-). These results strongly suggest that the small RNA that we detect is a tasiRNA whose generation depends on the function of RDR6.

To investigate the *TAS4* transcript levels in skin and flesh tissues Real-Time Quantitative Reverse Transcription polymerase chain reaction (RT-qPCR) was performed (Supplementary Figures S4A,B). In tuber skin high level of *TAS4* was detected in IVP48, Desiree and Congo cultivars. Tuber flesh of Desiree and Congo showed high levels of *TAS4* transcript compared to DB22670 and IVP48. RNA Ligase-Mediated (RLM)-Rapid Amplification of cDNA Ends (RACE) was performed in order to identify the cut site of miR828 on the *TAS4* transcript (Figure 4C). Several free 5′ ends of *TAS4* were detected with a predominant cut at the predicted cut site (between the 10th and 11th nucleotide of miR828). This result confirms that *TAS4* is processed by miR828 and is a direct target of the miRNA.

### miR828 Accumulation Is Coupled With Purple Color in the Same Tuber

**FIGURE 5:**
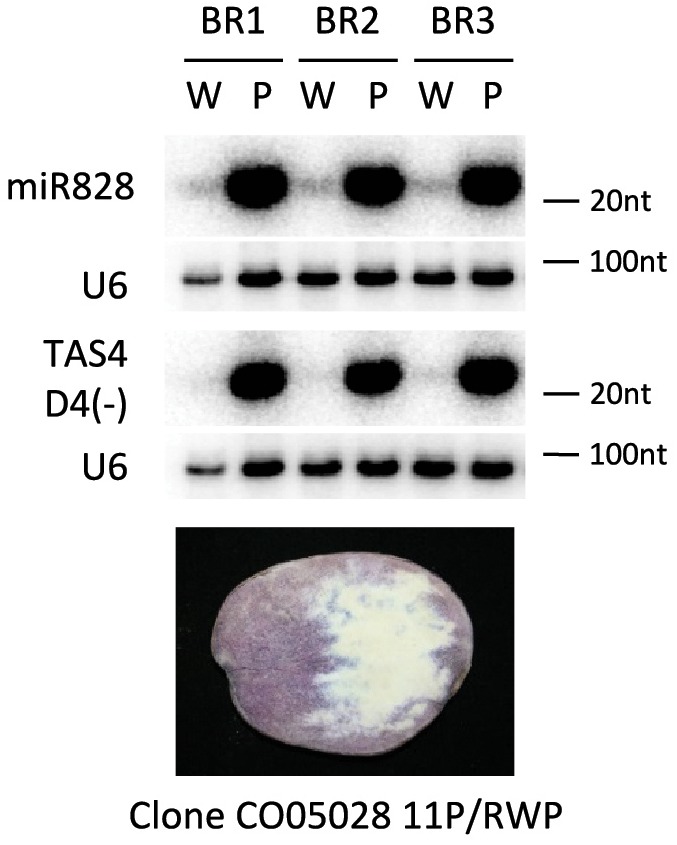
RNA gel blot analysis of small RNAs. Purple (P) and white (W) sectors of clone CO05028 11P/RWP were investigated for the presence of miR828 and TAS4 D4(-) with radioactively labeled probes specific to the small RNAs; U6 was used as a loading control. BR indicates biological replicates.

In the previous experiments different potato cultivars were investigated for the presence of miR828. Potato is a highly heterozygous crop; studying the genomes of 83 tetraploid cultivars it was shown that there was one single nucleotide polymorphism (SNP) in 24 base pair (bp) on average when exons were investigated and the frequency of SNPs was even higher in introns: 1 SNP per 15 bp (Potato Genome and Sequencing Consortium, 2011; Uitdewilligen et al., 2013). This suggests that there might be considerable differences in the genetic background of the cultivars we have studied. In order to decrease the confounding genetic, environmental and developmental effect we might face comparing different cultivars, we studied the expression of miR828 and TAS4 D4(-) in an advanced potato clone which was developed by conventional breeding procedures (Stushnoff et al., 2010). Clone CO05028 11P/RWP was chosen because it produces tubers with incomplete expression of tuber flesh pigmentation. Lyophilised tuber slices were used to isolate purple and white sectors of tuber flesh. The isolated material was subjected to RNA isolation and small RNA gel blot analysis was used to investigate miR828 expression level (Figure 5). We found that miR828 is expressed at a high level in the purple pigmented sectors but very low level of miR828 was detected in non-pigmented sectors. Similarly to miR828, TAS4 D4(-) was very abundant in purple sectors of tuber flesh and hardly detectable in the non-pigmented sectors. *TAS4* transcript level showed similar pattern to the cultivars having high expression in the purple sectors of the flesh (Supplementary Figure S4C). These data show that high expression of miR828 is coupled with high levels of TAS4 D4(-) in the same tuber and is associated with the presence of purple tuber flesh.

### Expression of *MYB* Genes in Tuber Tissues

**FIGURE 6:**
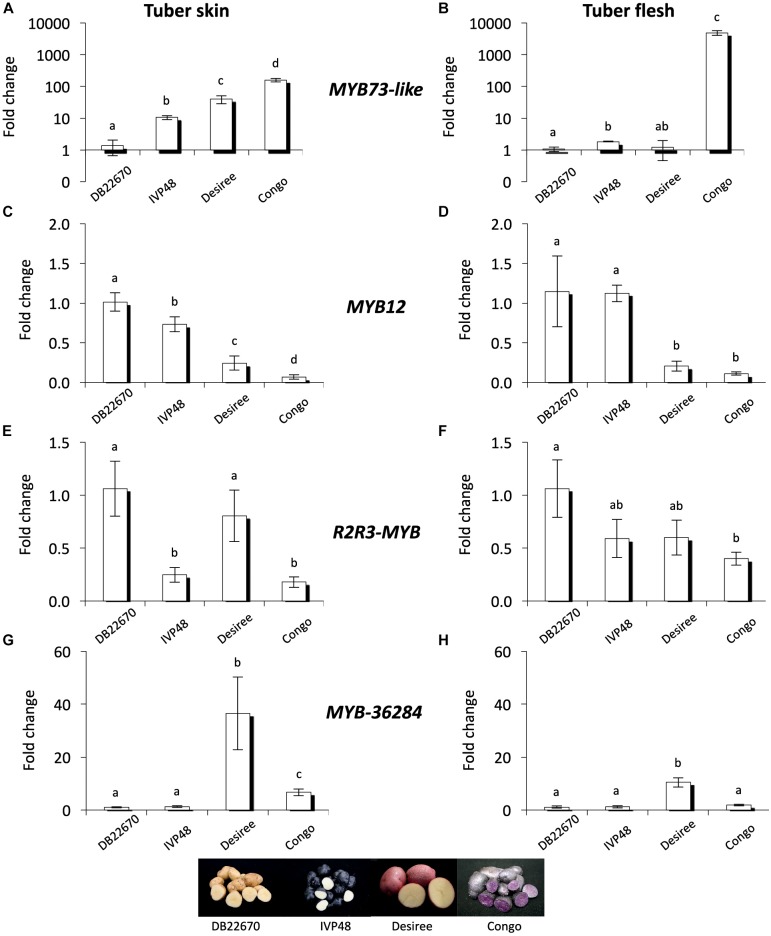
Expression analysis of MYB genes in cultivars. In tuber skin and flesh samples RT-qPCR was used to analyze the expression of *MYB73-like* **(A)** and **(B)**; *MYB12* **(C)** and **(D)**; *R2R3-MYB* **(E)** and **(F)** and *MYB-36284* **(G)** and **(H)** genes. Relative quantification was made to DB22670; the letters on the bars indicate significantly different expression levels of genes using Student’s *T*-test (*p* < 0.05); different letters mean significant differences between the samples.

MYB genes control anthocyanin formation in plants (Jung et al., 2009; Jaakola, 2013; Rock, 2013). The expression of a *MYB73-like* gene (a single domain MYB transcription factor) showed strong association with purple pigmented sectors of tuber flesh in advanced potato cultivars (Stushnoff et al., 2010). The expression level of *MYB73-like* gene was investigated by RT-qPCR in cultivars with different tuber skin and flesh colors (Figure 6). *MYB73-like* transcripts showed higher accumulation levels in skin tissues of IVP48, Desiree and Congo cultivars (deep blue, red, and deep purple colors, respectively) compared to DB22670 skin (yellow color). We detected the highest expression level in Congo skin (Figure 6A). A similar association was found in tuber flesh samples; only Congo flesh has purple color and extremely high expression (>1000-fold over DB22670) was detected for the *MYB73-like* gene. DB22670, IVP48, and Desiree (white and yellow flesh colors) all showed low levels of *MYB73-like* gene expression with only IVP48 flesh showing slightly elevated expression (Figure 6B). Altogether, we found that the *MYB73-like* gene had higher expression in purple tuber tissues but not only in flesh but also in skin which is in line with the previous findings (Stushnoff et al., 2010).

In order to investigate the potential target genes of miR828 and TAS4 D4(-) we used psRNATarget and the potato transcriptome database to predict target RNAs (Dai and Zhao, 2011; Potato Genome and Sequencing Consortium, 2011). Predicted targets include MYB transcription factors (Supplementary Table S2) and their expression levels were investigated by RT-qPCR. *MYB12* (PGSC0003DMT400018841) was predicted to be targeted by miR828 and the mRNA is degraded through cleavage. *MYB12* showed the highest accumulation level in DB22670 skin tissues (yellow) (Figure 6C). We found decreased expression levels in IVP48 (deep blue), Desiree (red) and Congo (deep purple) skin; lower *MYB12* expression is associated with anthocyanin accumulation and higher expression of miR828 (Figure 2B). In tuber flesh *MYB12* showed higher expression levels in DB22670 and IVP48 which have white flesh (Figure 6D). In contrast, Desiree and Congo flesh has lower amounts of *MYB12* transcript. Interestingly, Desiree has yellow flesh color and low amount of miR828 accumulates in flesh but it contains as low a level of *MYB12* as the purple Congo flesh. The inverse expression levels of miR828 and *MYB12* might suggest that miR828 can target *MYB12*.

Another predicted target of miR828 is an *R2R3-MYB* transcription factor (PGSC0003DMT400029235). The expression level of *R2R3-MYB* gene showed higher levels in DB22670 and Desiree skin (yellow and red colors, respectively) (Figure 6E). Compared to their expression, *R2R3-MYB* has lower expression in IVP48 and Congo skin samples (deep blue and purple). Although, miR828 expression was higher in Desiree skin (red color) (Figure 2B), *R2R3-MYB* expression was as high in Desiree skin as in DB22670 skin samples (yellow). In flesh tissues, *R2R3-MYB* showed different expression levels compared to skin samples; differential expression was only found between DB22670 and Congo (yellow and purple colors, respectively) (Figure 6F). Congo flesh tissue showed the lowest expression level of *R2R3-MYB* which is purple compared to flesh samples of DB22670 (yellow), IVP48 (white), and Desiree (yellow). It is notable that there is an increased expression of *R2R3-MYB* in IVP48 and Desiree flesh samples but it is not significantly different compared to Congo flesh. The accumulation levels of miR828 and *R2R3-MYB* suggest that there is a possibility that miR828 could target *R2R3-MYB*.

It is predicted that TAS4 D4(-) can also target an unannotated MYB transcription factor (PGSC0003DMT400036284, named as *MYB-36284* in this study) in potato. Its closest ortholog in *A. thaliana* is MYB113 (AT1G66370) which regulates the anthocyanin biosynthesis pathway and is targeted by miR828 and TAS4 D4(-) as well (Luo et al., 2012). The expression level of *MYB-36284* was investigated by RT-qPCR in skin tissues (Figure 6G) and was found to be similar in DB22670 (yellow) and IVP48 (deep blue) skin samples. Elevated expression was detected in Desiree (red) and Congo (deep purple) skins, being significantly lower in Congo compared to Desiree. A similar expression level of *MYB-36284* was found in flesh tissues having significantly higher expression in Desiree flesh (yellow) only compared to DB22670 (yellow), IVP48 (white) and Congo (purple) flesh samples (Figure 6H). Although, the expression pattern between TAS4 D4(-) and *MYB-36284* does not support miRNA directed regulation (no inverse correlation) we cannot exclude the possibility that the tasiRNA can target *MYB-36284*.

**FIGURE 7:**
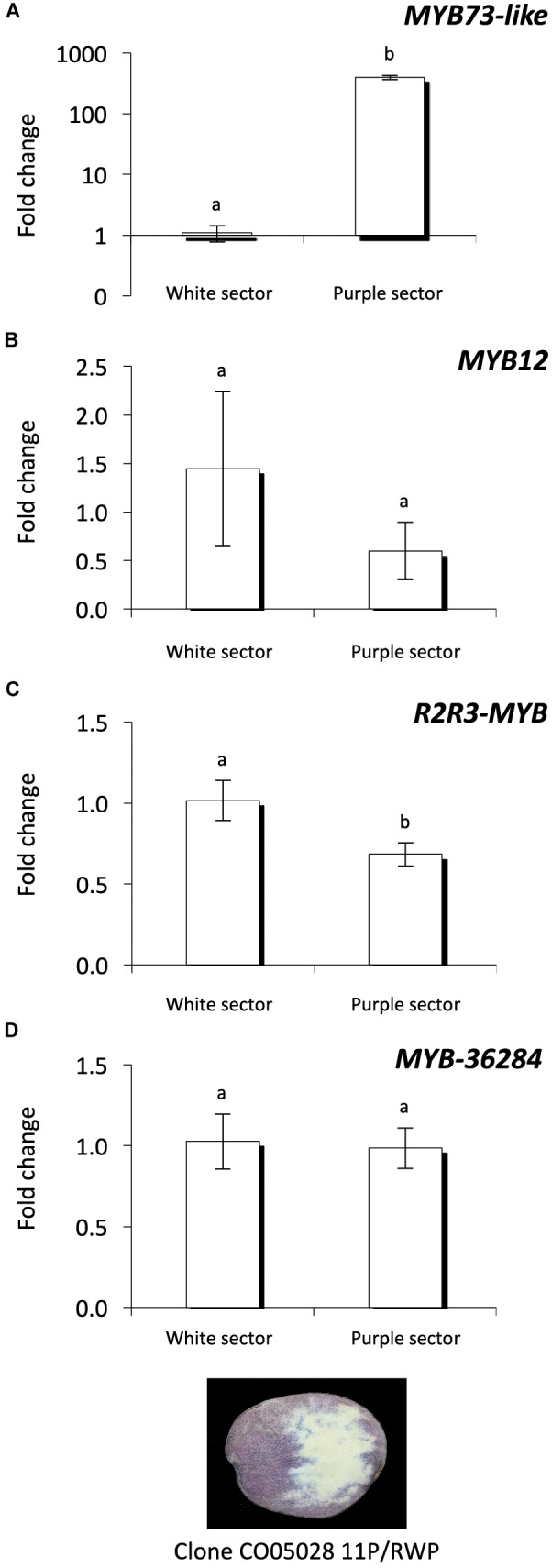
Expression analysis of MYB genes in the same tuber. Purple and white sectors of CO05028 11P/RWP were used to analyze the expression of *MYB73-like* **(A)**; *MYB12* **(B)**; *R2R3-MYB* **(C)**; and *MYB-36284* **(D)** genes in tuber flesh. Relative quantification was made to white sector samples; the letters on the bars indicate significantly different expression levels of genes using Student’s *T*-test (*p* < 0.05); different letters mean significant differences between the samples.

Investigating the purple and white sectors of the CO05028 11P/RWP clone we found that *MYB73-like* gene expression level is much higher in the purple tuber flesh sectors compared to the white ones similar to the expression levels detected in the cultivars (Figure 7A). This is in line with previous findings (Stushnoff et al., 2010). *MYB12* transcription factor showed high variation among the white sector samples but the expression of the gene was not significantly different compared to the purple sectors of the tubers (Figure 7B). In contrast, *R2R3-MYB* had significantly lower levels in purple sectors compared to the white tissues of the same tubers (Figure 7C) similar to the cultivars where *R2R3-MYB* showed lower expression in purple tissues (Figures 6E,F). *MYB-36284* does not show differential expression between the white and purple sectors of the tubers (Figure 7D). Altogether, we found similar patterns of the investigated small targets in white and purple sectors of the same tubers but with some differences compared to the cultivars.

### MYB Transcription Factors Are Direct Targets of Small RNAs in Potato

**FIGURE 8:**
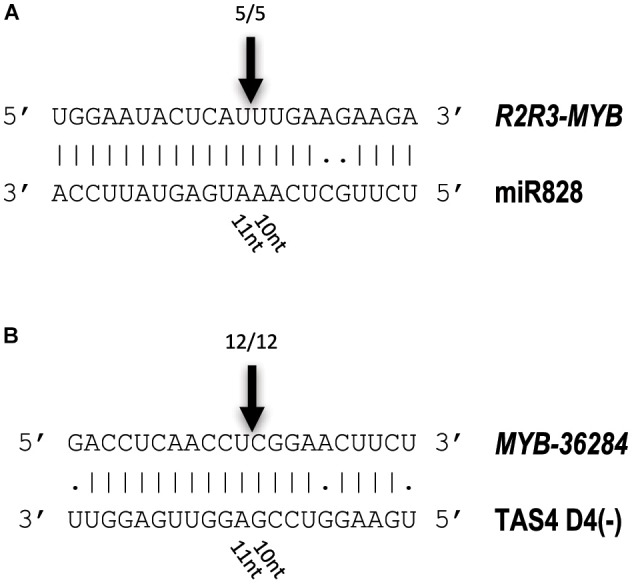
Identification of free 5′ ends of target RNAs. RLM 5′ RACE was used to identify free ends of *R2R3-MYB* **(A)** and *MYB-36284* **(B)** mRNAs. Arrows and numbers indicate free RNA ends with the frequency of cut points identified; the cut occurs between 10/11 nucleotides of the small RNAs (canonical cut site).

To investigate whether the predicted MYB transcription factors are direct targets of miR828 and TAS4 D4(-), RNA Ligase-Mediated (RLM)-Rapid Amplification of cDNA Ends (RACE) was performed using Congo skin samples. The free end of the *R2R3-MYB* mRNA was found and mapped back to one point of the mRNA (Figure 8A). The cut site was identified to the position where the miRNA cut usually occurs, between the 10/11 nucleotide positions of miR828 confirming that the *R2R3-MYB* mRNA is a direct target of miR828. A similar result was obtained for the *MYB-36284* transcript. The free end of the mRNA was found using RLM 5′ RACE which was mapped to the canonical cut site for the small RNA between the position of 10/11 nucleotides (Figure 8B). This finding confirms that *MYB-36284* mRNA is subjected to nuclease activity and it is a direct target of TAS4 D4(-). Although attempted several times, we could not identify free ends of *MYB12* using RLM 5′ RACE which indicates that this mRNA might not be a direct target of miR828.

## Discussion

Anthocyanins are health promoting bioactive compounds having increasing importance in human diet. The presence of anthocyanins could make fruits, tubers and other products more attractive for consumers and increases their values. The biosynthetic pathway and the genetic regulation of anthocyanin production are relatively well known but there is a gap in our understanding of the post-transcriptional regulation of anthocyanin biosynthesis. In this study, we investigated the role of short non-coding RNAs regulating MYB transcription factors of anthocyanin production and found a strong association between the presence of specific small RNAs and purple color in potato tuber skin and flesh. miR828 in potato has been characterized for the first time and we show that it initiates the production of tasiRNAs in different tissues increasing our knowledge of the fine regulation of gene expression at post-transcriptional level in this important crop. Additionally, we determined direct targets of the small RNAs which could be candidate genes for future potato improvement contributing global food security around the world.

### Small RNA Target Sites Are Conserved for miR828 and TAS4 D4(-)

Potato is a highly heterozygous plant having a high frequency of SNPs in exons and introns (Uitdewilligen et al., 2013). Additionally, many cultivars are tetraploid making it more difficult to conduct genetics and molecular biology studies (Potato Genome and Sequencing Consortium, 2011). Performing experiments in potato, we always have to consider that there is a possibility of having many different alleles of the same genes. This diversity emphasizes the importance of the role of small RNA directed gene regulation which can generally target many alleles of the same gene. However, this regulation needs the regulatory (target) sites to be conserved in order to establish small RNA directed gene silencing (Jones-Rhoades, 2012). We have performed expression analysis of chosen target genes in order to determine their steady-state levels in tuber skin or flesh tissues (Figures 6, 7). However, it is essential to know whether the small RNAs can direct post-transcriptional processing of their target genes at the predicted small RNA binding sites. To investigate the nucleotide composition of target sites we have performed sequencing of the PCR amplicons to detect any SNPs which might occur in the small RNA cut sites in all the investigated cultivars (Supplementary Figure S5). The lack of SNPs was verified in all cultivars for all of the genes which were investigated showing that the small RNA recognition sites are conserved among these plants (data not shown) and allows the action of effector complexes. This finding further supports the idea that the small RNA regulation of the investigated MYB transcription factors is conserved and could influence regulators of the anthocyanin biosynthesis pathway.

### Anthocyanin Level Correlates With Small RNA Expression

Model plants and crops including members of the *Solanaceae* family were investigated for anthocyanin content (Holton and Cornish, 1995; Dhar et al., 2015). Although, in arabidopsis it was found that the anthocyanin content of plants over-expressing miR828 was decreased and its target genes repressed (Yang et al., 2013) we found in potato that increased miR828 and TAS4 D4(-) levels are associated with anthocyanin accumulation in tuber skin and flesh tissues (Figure 3). Similar results were found in *A. thaliana* when exogenous sucrose and glucose were provided to seedlings (Luo et al., 2012). Increased sucrose level had a positive effect on TAS4 D4(-) expression and in parallel increased anthocyanin accumulation was observed in plants similar to our observation that the high levels of TAS4 D4(-) is associated with high levels of anthocyanins in tuber skin and flesh. miR156 targets *SPL9* in *A. thaliana* which negatively regulates anthocyanin production (Gou et al., 2011). Anthocyanins accumulate to the highest level at the junction between rosette and stem. High miR156 level promotes anthocyanin biosynthesis through the negative regulation of *SPL9* gene because SPL9 can destabilize the MYB-bHLH-WD40 transcriptional activation complex. These examples show that small RNAs can target negative regulators of anthocyanin biosynthesis ultimately influencing the production of bioactive compounds.

### Negative Regulators of Anthocyanin Biosynthesis

A conserved network of activators and repressors regulating anthocyanin pigmentation was identified in eudicots highlighting the complexity of this biosynthetic pathway (Albert et al., 2014; Smita et al., 2015). In our study we have investigated the expression levels of *MYB73-like* gene as a positive regulator of anthocyanin synthesis; its expression correlated well with the presence of anthocyanins (Figures 6, 7). However, it is not well known how the interaction of activators and repressors can contribute to the final color appearance. Our study dissects the role of repressors of anthocyanin biosynthesis contributing to the understanding of the balance of these factors. Repressors are especially important to fine-tune the production of anthocyanins. In petunia it was found that MYB27, an R2R3-MYB transcription factor, is an anthocyanin repressor (Albert et al., 2014). Over-expressing MYB27 reduced the pigmentation of petunia flowers. MYB27 targets both the anthocyanin pathway genes and basic-helix-loop-helix (bHLH) *ANTHOCYANIN1* (*AN1*) which is an essential component of the MYB-bHLH-WD repeat (MBW) activation complex. In the model legume *Medicago truncatula* MYB2 was shown to be a repressor of anthocyanins and proanthocyanidins (Jun et al., 2015). Over-expression of MYB2 reduced anthocyanin accumulation in *M. truncatula* hairy roots and *A. thaliana* seeds. Additionally, *myb2* mutant seedlings displayed expanded anthocyanin deposition in seedlings and flowers indicating a repressor function of MYB2 in anthocyanin production. In *Trifolium repens* *Tr-MYB133* and *Tr-MYB134* were identified as R2R3-MYB repressors of anthocyanin production (Albert, 2015). Interestingly, they do not prevent ectopic accumulation of anthocyanins and proanthocyanidins and they are expressed when these molecules are synthesized. These genes are regulated by MBW complexes and provide feedback regulation for the same complexes establishing a feedback regulatory network in legumes. In tetraploid potato cultivars R2R3-MYB transcription factors were extensively studied (Liu et al., 2016). *StMYB113* and *StMYBA1* were highly expressed in white tuber skins and lower expression levels in red or purple tissues. Several alleles of these genes were identified and functional analysis in tobacco leaves confirmed that the presence of a C-terminal 10-amino acid motif is important for activating anthocyanin accumulation suggesting a positive role of these genes in anthocyanin production. However, some variants of these genes were well expressed even in the absence of pigmentation which may be due to non-functional version of the proteins or inhibitory effect on anthocyanin production.

In this study we found that miR828 and TAS4 D4(-) are highly expressed in pigmented tissues of potato tubers (Figure 3) which implies opposite functions of their target genes in tissue color formation, being probably negative regulators. The small RNAs showed the same pattern not only in different cultivars but in white and purple sectors of the same tubers (Figure 5). At the same time the investigated targets of miR828 showed inverse correlation to the miRNA (Figures 6, 7) suggesting that the reduction of the expression of *MYB12* and *R2R3-MYB* might influence anthocyanin accumulation in both skin and flesh tissues. This expression pattern strongly suggests a negative role for these genes in anthocyanin production/accumulation being probably a repressor of pigmentation similarly to the MYB repressors which were discussed previously (Albert et al., 2014; Smita et al., 2015). *MYB-36284* is a direct target of TAS D4(-) (Figure 8). We could not detect differential expression of *MYB-36284* in pigmented and non-pigmented tissues either in cultivars or in the differentially pigmented sectors of the same tuber (Figures 6, 7). But it is highly possible that the tasiRNA regulation of the gene is important to keep *MYB-36284* expression at the level which can fulfill the role of the gene. During tomato fruit ripening it was shown that about half of the investigated miRNA-mRNA expression does not match the expected inverse correlation (Lopez-Gomollon et al., 2012). The reason detecting different small RNA levels and similar accumulation of *MYB-36284* in the same tissues could be that this process is spatio-temporally regulated. During tissue development and anthocyanin production/accumulation it might be that *MYB-36284* has an important role and its expression level is changed according to the biosynthetic pathway. Also, the fine regulation of *MYB-36284* by TAS4 D4(-) might be an important component to establish the balance between positive and negative regulators of anthocyanin biosynthesis.

**FIGURE 9:**
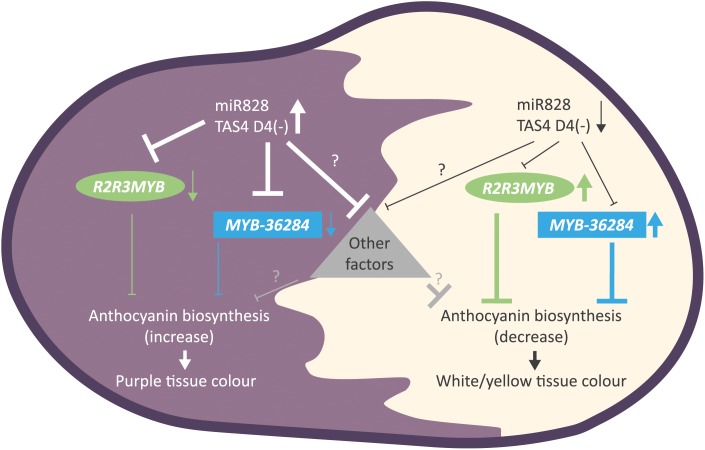
Summary of the findings for small RNA directed gene regulation of anthocyanin biosynthesis in potato. Purple tuber skin and flesh tissues show high levels of miR828 and TAS4 D4(-) and reduced expression of MYB genes (*R2R3MYB*, *MYB-36284*). The down-regulation of anthocyanin biosynthesis inhibitors results in the appearance of purple color. In contrast, white and yellow tissues have low accumulation levels of small RNAs allowing higher expression of MYB genes (*R2R3MYB*, *MYB-36284*). High levels of MYB transcription factors inhibits anthocyanin biosynthesis in skin and flesh tissues resulting in white or yellow color formation. Other, yet unknown factors might influence color development regulated directly or indirectly by small RNAs represented as a triangle.

This study explores the role of post-transcriptional regulation of gene expression in tissue color formation in potato and is summarized in Figure 9. Our results suggest the existence of the balance between activators and repressors of anthocyanin biosynthesis which is influenced by a newly identified level: not only the orchestration of transcription plays a crucial role but the expression levels of negative regulators are fine-tuned via small RNA regulation. In order to clarify the role of miR828 and TAS4 D4(-) in anthocyanin production further studies must be performed. In RDR6i transgenic Desiree lines we could not detect color change of tuber skins and flesh (data not shown). This indirectly indicates that TAS4 D4(-) might not be involved in anthocyanin regulation but to decide this TAS4 D4(-) over-expression and depletion studies must be performed. Similarly, miR828 could be over-expressed in cultivars with white skin and flesh or depleted in potato plants bearing purple/red tubers in order to prove the direct role of miR828 in anthocyanin regulation. Moreover, the role of the investigated *MYB* genes could be studied via a transgenic approach taking into account the small RNA regulation; small RNA cleavage resistant versions of mRNAs could be over-expressed or the genes could be silenced via different approaches to shed light on their role in anthocyanin biosynthesis.

## Materials and Methods

### Plant Material

*Solanum tuberosum* L. ‘Desiree’ and ‘Congo’ cultivars, DB22670 and IVP48 (Wilkinson et al., 1995) (*S. tuberosum* group *Phureja*) were used for the experiments. Plants were propagated from seed tubers for 8 weeks in a glasshouse under LD (16 h light/8 h dark) conditions in 38 cm wide pots. Leaves (2–6 from shoot) were collected together; tubers were harvested 4 months after planting, skin tissues were collected separately from flesh using three tubers from the same plant as one biological replicate.

### Generation of RDR6i Lines

The sequence of potato RDR6 (PGSC0003DMT400060673) was used to amplify a 330 nt long region of RDR6 in Desiree. The Gateway cloning system was used with primers having appropriate sequence (Supplementary Table S3) in order to clone the fragment into pDONR207 using BP clonase II (Invitrogen, United States). The cloned RDR6 sequence was confirmed by Sanger sequencing and cloned into the pBIN19RNAi vector using LR Clonase II Enzyme mix according to the manufacturer’s instruction (Invitrogen, United States). The AGL1 strain of agrobacterium was used to generate transgenic *S. tuberosum* L. ‘Desiree’ lines expressing the hairpin construct using standard transformation procedures (Millam, 2007). Kanamycin was used as a selection marker to establish RDR6i lines. The expression of the endogenous RDR6 gene was studied using primers (Supplementary Table S3, RDR6_UPL) outside of the region which was used to generate the hairpin. UPL Probe #42 was used with the following conditions for amplification: 10 min denaturation at 95°C followed by 40 cycles of 15 s at 95°C, 60 s at 60°C. For more details of PCR amplification, please see Quantitative RT-PCR description.

For transient assay, 3 weeks old *N. benthamiana* plant were used and infiltrated with the RDR6i construct and empty vector (pBIN19RNAi) as it was described in Wypijewski et al. (2009). Total RNA was extracted and small RNAs were analyzed as it is described below.

### Bioinformatics Analysis of Small RNAs

Total RNA for small RNA library preparation was made using the Plant/Fungi Total RNA Purification Kit (Norgen Biotek, Canada) according to the manufacturer’s suggestion. Small RNA library preparation and sequencing was performed by BaseClear (Netherlands); the libraries were sequenced using HiSeq2000 as single end, short (36 cycles) reads. Bioinformatics analysis of small RNAs including miRNA prediction was performed as described in Zhang et al. (2013). Secondary pre-miRNA structure of miR828 was predicted by Mfold and the lowest free energy is shown in Figure 1 (Zuker, 2003). Target RNA prediction of miR828 and TAS4 D4(-) was performed using psRNATarget online software (V1 scoring schema, 2011 release) (Dai and Zhao, 2011). The description of parameters used for psRNATarget prediction is described in Zhang et al. (2013).

### RNA Analysis and Identification of miRNA Cut Sites

Total RNA was extracted from leaf, tuber skin and flesh material using TRI Reagent (Sigma-Aldrich, United States) according to the manufacturer’s instructions.

For RNA gel blot hybridization of small RNAs total RNA (10 μg for leaf, 5 μg for skin and flesh tissues) was separated by 15% polyacrylamide (19:1) gel with 8 M urea and 1 × MOPS (20 mM MOPS/NaOH, pH7) buffer. RNA markers (Decade RNA markers, Ambion, United States) were end labeled by 32γ-ATP according to the manufacturer’s instructions. RNA was blotted onto Hybond-N membrane (Amersham, GE Healthcare, United Kingdom) using a Panther^TM^ Semi-dry Electroblotter, HEP-1 (Thermo Scientific Owl Separation Systems, United States) and cross-linked by *N*-(3-Dimethylaminopropyl)-*N*′-ethylcarbodiimide hydrochloride (EDC, Sigma-Aldrich, United States) (Pall et al., 2007). DNA oligos (20 pmol) were end-labeled by 32γ-ATP using T4 polynucleotide kinase (NEB, United States) to visualize miRNAs. For sequences see Supplementary Table S3. Hybridization was performed in PerfectHyb^TM^ Plus Hybridization Buffer (Sigma-Aldrich, United States). After overnight incubation at 37°C, the membrane was washed twice in 2X SSC and 0.1% SDS for 15 min at 37°C. After washing, membranes were exposed to a phosphorimager plate and signals were detected by the FLA-7000 Fluorescent Image Analyzing System and Aida Image Analyzer software (Fujifilm, United States). To visualize RDR6 derived siRNAs, random DNA probe was prepared using the fragment which was used to clone the RNAi construct by Prime-a-Gene^®^ Labeling System (Promega, United States) according to the manufacturer’s instruction. The probe was used as described above.

For the identification of miRNA cut sites, RNA Ligase-Mediated (RLM)-Rapid Amplification of cDNA Ends (RACE) was performed using the GeneRacer kit (Invitrogen, United States) according to the manufacturer’s instructions. 5′ RNA oligo was ligated to mRNAs which was isolated using 130 μg total RNA originating from Congo skin tissue with PolyATtract mRNA Isolation Systems (Promega, United States) according to the manufacturer’s instructions. Platinum Taq polymerase (Invitrogen, United States) was used for the amplification of genes with kit primers and gene specific primers (Supplementary Table S3); isolated DNA fragments were ligated into pGEM-T Easy vector system (Promega, United States) for sequence analysis. Sequencing was performed using ABI 3730 DNA Analyzer (Applied Biosystems, Thermo Fisher Scientific, United States) and CLC main workbench (Qiagen, United States) was used for the analysis of the results.

### Quantitative RT-PCR

One microgram of total RNA was used to prepare cDNA in a reverse transcription reaction (MMLV, Promega, United States) according to the manufacturer. Three technical replicates of each three biological replicates were used in subsequent Real-Time Quantitative Reverse Transcription polymerase chain reaction (RT-qPCR) reactions using FastStart Universal Probe Master with Rox (Roche Diagnostics, Switzerland) for the control gene: *StEF1a* with probe #117. The Universal Probe Library system was used to design primers; its description and the details of RT-qPCR are described in Campbell et al. (2014). A StepOnePlus Real-Time PCR System (Applied Biosystems, Thermo Fisher Scientific, United States) was used for amplification with the following conditions: 10 min denaturation at 95°C followed by 40 cycles of 15 s at 95°C, 60 s at 59°C. PCR primer sets were designed flanking the miR828 recognition sites on the target mRNAs using Primer3 (Untergasser et al., 2012). RT-qPCR reactions were performed using Precision Fast 2xqPCR Mastermix with ROX (PrimerDesign, United Kingdom) with 2 min denaturation at 95°C followed by 40 cycles of 5 s at 95°C, 5 s at 58°C and 10 s at 72°C. Data was analyzed as described in Hornyik et al. (2010). For the expression analysis of target genes primers were designed flanking the miRNA cut region. The PCR amplicons of the studied genes were confirmed by sequencing using pGEM^®^-T Easy vector system (Promega, United States) and Sanger sequencing at the Genome Technology facility of The Hutton. The details of genes and primers are in Supplementary Table S3.

### Total Polyphenol and Anthocyanin Extraction and Analysis

Tuber skin and flesh tissues were sliced, frozen in liquid nitrogen, homogenized later using pestles and mortals under liquid nitrogen and aliquots were freeze-dried for downstream analysis.

For analysis, 100 ± 5 mg of freeze-dried potato powder was extracted with 2.5 ml of 50% (v/v) aqueous acetonitrile containing 0.2% formic acid in a blood rotator for 30 min in the dark at 4°C (Stushnoff et al., 2010). Tubes were centrifuged (2500 *g* for 10 min at 4°C) and the supernatant decanted. The extraction procedure was repeated on the residue. The combined supernatants were assayed for phenol content using the Folin and Ciocalteu method and the differential colorimetric method for anthocyanins (Deighton et al., 2000). Subsamples (1 mL) were dried using a Speed-Vac and re-suspended in 500 μl of 5% acetonitrile for liquid chromatography–mass spectrometry (LC–MS) analysis.

Samples were analyzed on an LCQ-Deca system, comprising Surveyor autosampler, pump, and photodiode array detector (PDAD) and a Thermo ion-trap mass spectrometer (Thermo Fisher Scientific, San Jose, CA, United States). The PDAD scanned discrete channels at 280, 365, and 520 nm. The samples were applied to a C18 column (Synergi Hydro C18 with polar endcapping, 2.0 mm × 250 mm, Phenomenex Ltd., Macclesfield, United Kingdom) and eluted using a linear gradient of 5% acetonitrile (0.1% formic acid) to 40% acetonitrile (0.1% formic acid) over 30 min at a rate of 200 μL/min. The LCQ-Deca LC–MS was fitted with an ESI (electrospray ionization) interface and analyzed the samples in positive and negative ion modes. There were two scan events; full scan analysis followed by data-dependent MS/MS of the most intense ions using collision energies (source voltage) of 45%. The capillary temperature was set at 250°C, with sheath gas at 60 psi and auxiliary gas at 15 psi. Peaks were identified by comparing their relative retention times, PDA spectra and mass-to-charge ratio (*m/z*) and MS^2^ properties with previous reports (Fossen et al., 2003; Eichhorn and Winterhalter, 2005; Stushnoff et al., 2008, 2010; Oertel et al., 2017) or, where available, against standard compounds (e.g., chlorogenic acid, tyrosine, and phenylalanine). Although not quantitative, this approach gives valid relative comparisons of the components between different samples or treatments. Samples were also run at 50% dilution to check that the relative amounts of the components were consistent.

### Statistical Analysis

Quantitative RT-PCR data was analyzed as described in Hornyik et al. (2010). Fold change in expression was calculated relative to DB22670 or the white sectors of clone CO05028 11P/RWP using *StEF1a* (PGSC0003DMT400059830) mRNA as a reference. Pairwise Student’s *t*-test (*p* < 0.05) was used to determine statistically different expression levels of the investigated genes; different letters on the RT-qPCR graphs show significantly different expression levels of genes. Total polyphenol and anthocyanin content (TPC and TAC, respectively) were statistically analyzed to compare samples; pairwise Student’s *T*-test (*p* < 0.05) was performed to show the significant differences between skin or flesh tissues. Components of anthocyanins were quantified by their m/z peak areas calculated using the software provided with the instrument and expressed as average ± standard errors.

## Data Availability

The small RNA dataset generated for this study can be found in the European Nucleotide Archive (ENA) under the study accession number PRJEB26603 (http://www.ebi.ac.uk/ena/data/view/PRJEB26603).

## Author Contributions

NB and ML propagated the plants. NB, ML, and CH carried out the molecular work. RZ did the high-throughput analysis of small RNAs. JD, DD, and JS performed plant transformation. CA and GM performed the metabolomics study. MT, GB, and CH were involved in planning the experiments and writing the manuscript.

## Conflict of Interest Statement

The authors declare that the research was conducted in the absence of any commercial or financial relationships that could be construed as a potential conflict of interest.

## Footnotes

1 http://www.fao.org

## References

[B1] Albert N. W. (2015). Subspecialization of R2R3-MYB repressors for anthocyanin and proanthocyanidin regulation in forage legumes. *Front. Plant Sci.* 6:1165. 10.3389/fpls.2015.01165 26779194 PMC4689181

[B2] Albert N. W. Davies K. M. Lewis D. H. Zhang H. B. Montefiori M. Brendolise C. (2014). A conserved network of transcriptional activators and repressors regulates anthocyanin pigmentation in eudicots. *Plant Cell* 26 962–980. 10.1105/tpc.113.122069 24642943 PMC4001404

[B3] Axtell M. J. Meyers B. C. (2018). Revisiting criteria for plant microRNA annotation in the era of big data. *Plant Cell* 30 272–284. 10.1105/tpc.17.00851 29343505 PMC5868703

[B4] Borevitz J. O. Xia Y. J. Blount J. Dixon R. A. Lamb C. (2000). Activation tagging identifies a conserved MYB regulator of phenylpropanoid biosynthesis. *Plant Cell* 12 2383–2393. 10.1105/tpc.12.12.2383 11148285 PMC102225

[B5] Borges F. Martienssen R. A. (2015). The expanding world of small RNAs in plants. *Nat. Rev. Mol. Cell Biol.* 16 727–741. 10.1038/nrm4085 26530390 PMC4948178

[B6] Brown C. R. Culley D. Bonierbale M. Amoros W. (2007). Anthocyanin, carotenoid content, and antioxidant values in native south American potato cultivars. *HortScience* 42 1733–1736.

[B7] Campbell R. Pont S. D. A. Morris J. A. McKenzie G. Sharma S. K. Hedley P. E. (2014). Genome-wide QTL and bulked transcriptomic analysis reveals new candidate genes for the control of tuber carotenoid content in potato (*Solanum tuberosum* L.). *Theor. Appl. Genet.* 127 1917–1933. 24965888 10.1007/s00122-014-2349-0

[B8] Chandrasekara A. Josheph Kumar T. (2016). Roots and tuber crops as functional foods: a review on phytochemical constituents and their potential health benefits. *Int. J. Food Sci.* 2016:3631647. 10.1155/2016/3631647 27127779 PMC4834168

[B9] Cho K. Cho K. S. Sohn H. B. Ha I. J. Hong S. Y. Lee H. (2016). Network analysis of the metabolome and transcriptome reveals novel regulation of potato pigmentation. *J. Exp. Bot.* 67 1519–1533. 10.1093/jxb/erv549 26733692 PMC4762390

[B10] Dai X. Zhao P. X. (2011). psRNATarget: a plant small RNA target analysis server. *Nucleic Acids Res.* 39(Web Server issue), W155–W159. 21622958 10.1093/nar/gkr319 PMC3125753

[B11] De Jong W. S. Eannetta N. T. De Jong D. M. Bodis M. (2004). Candidate gene analysis of anthocyanin pigmentation loci in the Solanaceae. *Theor. Appl. Genet.* 108 423–432. 10.1007/s00122-003-1455-1 14523517

[B12] Deighton N. Brennan R. Finn C. Davies H. V. (2000). Antioxidant properties of domesticated and wild Rubus species. *J. Sci. Food Agric.* 80 1307–1313. 10.1002/1097-0010(200007)80:9<1307::Aid-Jsfa638<3.0.Co;2-P

[B13] Dhar M. K. Sharma R. Koul A. Kaul S. (2015). Development of fruit color in Solanaceae: a story of two biosynthetic pathways. *Briefings Funct. Genom.* 14 199–212. 10.1093/bfgp/elu018 24916164

[B14] Dodds K. S. Long D. H. (1955). The inheritance of colour in diploid potatoes. *J. Genet.* 53 136–149. 10.1007/bf02981517 26849236

[B15] Eichhorn S. Winterhalter P. (2005). Anthocyanins from pigmented potato (*Solanum tuberosum* L.) varieties. *Food Res. Int.* 38 943–948. 10.1016/j.foodres.2005.03.011

[B16] Fei Q. Xia R. Meyers B. C. (2013). Phased, secondary, small interfering RNAs in posttranscriptional regulatory networks. *Plant Cell* 25 2400–2415. 10.1105/tpc.113.114652 23881411 PMC3753373

[B17] Fossen T. Ovstedal D. O. Slimestad R. Andersen O. M. (2003). Anthocyanins from a Norwegian potato cultivar. *Food Chem.* 81 433–437. 10.1016/S0308-8146(02)004739

[B18] Gou J. Y. Felippes F. F. Liu C. J. Weigel D. Wang J. W. (2011). Negative regulation of anthocyanin biosynthesis in *Arabidopsis* by a miR156-targeted SPL transcription factor. *Plant Cell* 23 1512–1522. 10.1105/tpc.111.084525 21487097 PMC3101539

[B19] Guan X. Y. Pang M. X. Nah G. Shi X. L. Ye W. X. Stelly D. M. (2014). miR828 and miR858 regulate homoeologous MYB2 gene functions in *Arabidopsis* trichome and cotton fibre development. *Nat. Commun.* 5:3050. 10.1038/ncomms4050 24430011

[B20] Hancock R. D. Morris W. L. Ducreux L. J. Morris J. A. Usman M. Verrall S. R. (2014). Physiological, biochemical and molecular responses of the potato (*Solanum tuberosum* L.) plant to moderately elevated temperature. *Plant Cell Environ.* 37 439–450. 10.1111/pce.12168 23889235

[B21] Holton T. A. Cornish E. C. (1995). Genetics and biochemistry of anthocyanin biosynthesis. *Plant Cell* 7 1071–1083. 10.1105/tpc.7.7.1071 12242398 PMC160913

[B22] Hornyik C. Terzi L. C. Simpson G. G. (2010). The spen family protein FPA controls alternative cleavage and polyadenylation of RNA. *Dev. Cell* 18 203–213. 10.1016/j.devcel.2009.12.009 20079695

[B23] Hsieh L. C. Lin S. I. Shih A. C. C. Chen J. W. Lin W. Y. Tseng C. Y. (2009). Uncovering small RNA-mediated responses to phosphate deficiency in *Arabidopsis* by deep sequencing. *Plant Physiol.* 151 2120–2132. 10.1104/pp.109.147280 19854858 PMC2785986

[B24] Jaakola L. (2013). New insights into the regulation of anthocyanin biosynthesis in fruits. *Trends Plant Sci.* 18 477–483. 10.1016/j.tplants.2013.06.003 23870661

[B25] Jemison J. M. Sexton P. Camire M. E. (2008). Factors influencing consumer preference of fresh potato varieties in maine. *Am. J. Potato Res.* 85 140–149. 10.1007/s12230-008-9017-3

[B26] Jia X. Y. Shen J. Liu H. Li F. Ding N. Gao C. Y. (2015). Small tandem target mimic-mediated blockage of microRNA858 induces anthocyanin accumulation in tomato. *Planta* 242 283–293. 10.1007/s00425-015-2305-5 25916310

[B27] Jones-Rhoades M. W. (2012). Conservation and divergence in plant microRNAs. *Plant Mol. Biol.* 80 3–16. 10.1007/s11103-011-9829-2 21996939

[B28] Jun J. H. Liu C. G. Xiao X. R. Dixon R. A. (2015). The transcriptional repressor MYB2 regulates both spatial and temporal patterns of proanthocyandin and anthocyanin pigmentation in *Medicago truncatula*. *Plant Cell* 27 2860–2879. 10.1105/tpc.15.00476 26410301 PMC4682322

[B29] Jung C. S. Griffiths H. M. De Jong D. M. Cheng S. Bodis M. Kim T. S. (2009). The potato developer (D) locus encodes an R2R3 MYB transcription factor that regulates expression of multiple anthocyanin structural genes in tuber skin. *Theor. Appl. Genet.* 120 45–57. 10.1007/s00122-009-1158-3 19779693 PMC2778721

[B30] Jung C. S. Griffiths H. M. De Jong D. M. Cheng S. P. Bodis M. De Jong W. S. (2005). The potato P locus codes for flavonoid 3’,5’-hydroxylase (vol 110, pg 269, 2005). *Theor. Appl. Genet.* 111 184–184. 10.1007/s00122-005-1987-7 15565378

[B31] Kozomara A. Griffiths-Jones S. (2014). miRBase: annotating high confidence microRNAs using deep sequencing data. *Nucleic Acids Res.* 42 D68–D73. 10.1093/nar/gkt1181 24275495 PMC3965103

[B32] Lakhotia N. Joshi G. Bhardwaj A. R. Katiyar-Agarwal S. Agarwal M. Jagannath A. (2014). Identification and characterization of miRNAome in root, stem, leaf and tuber developmental stages of potato (*Solanum tuberosum* L.) by high-throughput sequencing. *BMC Plant Biol.* 14:6. 10.1186/1471-2229-14-6 24397411 PMC3913621

[B33] Lee S. H. Oh S. H. Hwang I. G. Kim H. Y. Woo K. S. Woo S. H. (2016). Antioxidant contents and antioxidant activities of white and colored potatoes (*Solanum tuberosum* L.). *Prevent. Nutr. Food Sci.* 21 110–116. 10.3746/pnf.2016.21.2.110 27390727 PMC4935237

[B34] Liu R. Lai B. Hu B. Qin Y. H. Hu G. B. Zhao J. T. (2017). Identification of MicroRNAs and their target genes related to the accumulation of anthocyanins in litchi chinensis by high -throughput sequencing and degradome analysis. *Front. Plant Sci.* 7:2059. 10.3389/fpls.2016.02059 28119728 PMC5223483

[B35] Liu Y. Tikunov Y. Schouten R. E. Marcelis L. F. M. Visser R. G. F. Bovy A. (2018). Anthocyanin biosynthesis and degradation mechanisms in solanaceous vegetables: a review. *Front. Chem.* 6:52. 10.3389/fchem.2018.00052 29594099 PMC5855062

[B36] Liu Y. H. Lin-Wang K. Espley R. V. Wang L. Yang H. Y. Yu B. (2016). Functional diversification of the potato R2R3 MYB anthocyanin activators AN1, MYBA1, and MYB113 and their interaction with basic helix-loop-helix cofactors. *J. Exp. Bot.* 67 2159–2176. 10.1093/jxb/erw014 26884602 PMC4809278

[B37] Lopez-Gomollon S. Mohorianu I. Szittya G. Moulton V. Dalmay T. (2012). Diverse correlation patterns between microRNAs and their targets during tomato fruit development indicates different modes of microRNA actions. *Planta* 236 1875–1887. 10.1007/s00425-012-1734-7 22922939

[B38] Lotkowska M. E. Tohge T. Fernie A. R. Xue G. P. Balazadeh S. Mueller-Roeber B. (2015). The *Arabidopsis* transcription factor MYB112 promotes anthocyanin formation during salinity and under high light stress. *Plant Physiol.* 169 1862–1880. 10.1104/pp.15.00605 26378103 PMC4634054

[B39] Luo Q. J. Mittal A. Jia F. Rock C. D. (2012). An autoregulatory feedback loop involving PAP1 and TAS4 in response to sugars in *Arabidopsis*. *Plant Mol. Biol.* 80 117–129. 10.1007/s11103-011-9778-9 21533841 PMC3272322

[B40] Millam S. (2007). “Potato (*Solanum tuberosum* L.),” in *Agrobacterium Protocols*, ed. Wang K. (Berlin: Sringer), 25–35.

[B41] Mori M. Hayashi K. Ohara-Takada A. Watanuki H. Katahira R. Ono H. (2010). Anthocyanins from skins and fleshes of potato varieties. *Food Sci. Technol. Res.* 16 115–122. 10.3136/fstr.16.115

[B42] Oertel A. Matros A. Hartmann A. Arapitsas P. Dehmer K. J. Martens S. (2017). Metabolite profiling of red and blue potatoes revealed cultivar and tissue specific patterns for anthocyanins and other polyphenols. *Planta* 246 281–297. 28664422 10.1007/s00425-017-2718-4

[B43] Pall G. S. Codony-Servat C. Byrne J. Ritchie L. Hamilton A. (2007). Carbodiimide-mediated cross-linking of RNA to nylon membranes improves the detection of siRNA, miRNA and piRNA by northern blot. *Nucleic Acids Res.* 35:e60. 10.1093/nar/gkm112 17405769 PMC1885651

[B44] Peragine A. Yoshikawa M. Wu G. Albrecht H. L. Poethig R. S. (2004). SGS3 and SGS2/SDE1/RDR6 are required for juvenile development and the production of trans-acting siRNAs in *Arabidopsis*. *Genes Dev.* 18 2368–2379. 15466488 10.1101/gad.1231804 PMC522987

[B45] Potato Genome and Sequencing Consortium (2011). Genome sequence and analysis of the tuber crop potato. *Nature* 475 189–195. 10.1038/nature10158 21743474

[B46] Rajagopalan R. Vaucheret H. Trejo J. Bartel D. P. (2006). A diverse and evolutionarily fluid set of microRNAs in *Arabidopsis* *thaliana*. *Genes Dev.* 20 3407–3425. 10.1101/gad.1476406 17182867 PMC1698448

[B47] Rock C. D. (2013). Trans-acting small interfering RNA4: key to nutraceutical synthesis in grape development? *Trends Plant Sci.* 18 601–610. 10.1016/j.tplants.2013.07.006 23993483 PMC3818397

[B48] Rogers K. Chen X. (2013). Biogenesis, turnover, and mode of action of plant microRNAs. *Plant Cell* 25 2383–2399. 10.1105/tpc.113.113159 23881412 PMC3753372

[B49] Rommens C. M. Richael C. M. Yan H. Navarre D. A. Ye J. S. Krucker M. (2008). Engineered native pathways for high kaempferol and caffeoylquinate production in potato. *Plant Biotechnol. J.* 6 870–886. 10.1111/j.1467-7652.2008.00362.x 18662373

[B50] Saito K. Yonekura-Sakakibara K. Nakabayashi R. Higashi Y. Yamazaki M. Tohge T. (2013). The flavonoid biosynthetic pathway in *Arabidopsis*: structural and genetic diversity. *Plant Physiol. Biochem.* 72 21–34. 10.1016/j.plaphy.2013.02.001 23473981

[B51] Singh A. Saraf S. Dasgupta I. Mukherjee S. K. (2016). Identification and validation of a virus-inducible ta-siRNA-generating TAS4 locus in tomato. *J. Biosci.* 41 109–118. 26949093 10.1007/s12038-016-9590-4

[B52] Smita S. Katiyar A. Chinnusamy V. Pandey D. M. Bansal K. C. (2015). Transcriptional regulatory network analysis of MYB transcription factor family genes in rice. *Front. Plant Sci.* 6:1157. 10.3389/fpls.2015.01157 26734052 PMC4689866

[B53] Stushnoff C. Ducreux L. J. Hancock R. D. Hedley P. E. Holm D. G. McDougall G. J. (2010). Flavonoid profiling and transcriptome analysis reveals new gene-metabolite correlations in tubers of *Solanum tuberosum* L. *J. Exp. Bot.* 61 1225–1238. 10.1093/jxb/erp394 20110266 PMC2826661

[B54] Stushnoff C. Holm D. Thompson M. D. Jiang W. Thompson H. J. Joyce N. I. (2008). Antioxidant properties of cultivars and selections from the Colorado potato breeding program. *Am. J. Potato Res.* 85 267–276. 10.1007/s12230-008-9032-4

[B55] Uitdewilligen J. G. Wolters A. M. D’hoop B. B. Borm T. J. Visser R. G. van Eck H. J. (2013). A next-generation sequencing method for genotyping-by-sequencing of highly heterozygous autotetraploid potato. *PLoS One* 8:e62355. 10.1371/journal.pone.0062355 23667470 PMC3648547

[B56] Untergasser A. Cutcutache I. Koressaar T. Ye J. Faircloth B. C. Remm M. (2012). Primer3–new capabilities and interfaces. *Nucleic Acids Res.* 40:e115. 10.1093/nar/gks596 22730293 PMC3424584

[B57] Vaucheret H. (2006). Post-transcriptional small RNA pathways in plants: mechanisms and regulations. *Genes Dev.* 20 759–771. 16600909 10.1101/gad.1410506

[B58] Vazquez F. Vaucheret H. Rajagopalan R. Lepers C. Gasciolli V. Mallory A. C. (2004). Endogenous trans-acting siRNAs regulate the accumulation of *Arabidopsis* mRNAs. *Mol. Cell.* 16 69–79. 10.1016/j.molcel.2004.09.028 15469823

[B59] Wilkinson M. J. Bennett S. T. Clulow S. A. Allainguillaume J. Harding K. Bennett M. D. (1995). Evidence for somatic translocation during potato dihaploid induction. *Heredity* 74 146–151. 10.1038/hdy.1995.21 7706107

[B60] Wypijewski K. Hornyik C. Shaw J. A. Stephens J. Goraczniak R. Gunderson S. I. (2009). Ectopic 5’ splice sites inhibit gene expression by engaging RNA surveillance and silencing pathways in plants. *Plant Physiol.* 151 955–965. 10.1104/pp.109.139733 19666706 PMC2754638

[B61] Xia R. Zhu H. An Y. Q. Beers E. P. Liu Z. R. (2012). Apple miRNAs and tasiRNAs with novel regulatory networks. *Genome Biol.* 13:R47. 10.1186/gb-2012-13-6-r47 22704043 PMC3446319

[B62] Xie Z. X. Allen E. Wilken A. Carrington J. C. (2005). DICER-LIKE 4 functions in trans-acting small interfering RNA biogenesis and vegetative phase change in *Arabidopsis thaliana*. *Proc. Natl. Acad. Sci. U.S.A.* 102 12984–12989. 10.1073/pnas.0506426102 16129836 PMC1200315

[B63] Yang F. X. Cai J. Yang Y. Liu Z. B. (2013). Overexpression of microRNA828 reduces anthocyanin accumulation in *Arabidopsis*. *Plant Cell Tissue Organ Cult.* 115 159–167. 10.1007/s11240-013-0349-4

[B64] Yu B. Yang Z. Y. Li J. J. Minakhina S. Yang M. C. Padgett R. W. (2005). Methylation as a crucial step in plant microRNA biogenesis. *Science* 307 932–935. 10.1126/science.1107130 15705854 PMC5137370

[B65] Zhang R. X. Marshall D. Bryan G. J. Hornyik C. (2013). Identification and characterization of miRNA transcriptome in potato by high-throughput sequencing. *PLoS One* 8:e57233. 10.1371/journal.pone.0057233 23437348 PMC3578796

[B66] Zhang Y. F. Jung C. S. De Jong W. S. (2009). Genetic analysis of pigmented tuber flesh in potato. *Theor. Appl. Genet.* 119 143–150. 10.1007/s00122-009-1024-3 19363602 PMC2690854

[B67] Zheng Y. Wang Y. Wu J. Ding B. Fei Z. J. (2015). A dynamic evolutionary and functional landscape of plant phased small interfering RNAs. *BMC Biol.* 13:32. 10.1186/s12915-015-0142-4 25980406 PMC4457045

[B68] Zuber T. Holm D. Byrne P. Ducreux L. Taylor M. Kaiser M. (2015). Optimization of in vitro inhibition of HT-29 colon cancer cell cultures by *Solanum tuberosum* L. extracts. *Food Funct.* 6 72–83. 10.1039/c4fo00649f 25338312

[B69] Zuker M. (2003). Mfold web server for nucleic acid folding and hybridization prediction. *Nucleic Acids Res.* 31 3406–3415. 12824337 10.1093/nar/gkg595 PMC169194

