# Supplementary material for: Potato miR828 Is Associated With Purple Tuber Skin and Flesh Color
Source: Front Plant Sci. 2018 Dec 11;9:1742. doi: 10.3389/fpls.2018.01742 (PMC6297172; doi:10.3389/fpls.2018.01742)
Supplement: Supplementary file 1 [file Data_Sheet_1.docx]

Supplementary Material

Potato miR828 is associated with purple tuber skin and flesh colour

Nicola Bonar^1^, Michele Liney^1§^, Runxuan Zhang^2^, Ceri Austin^3^, Jimmy Dessoly^1^, Diane Davidson^1^, Jennifer Stephens^1^, Gordon McDougal^3^, Mark Taylor^1^, Glenn J. Bryan^1^ and Csaba Hornyik^1*^

*** Correspondence:** Csaba Hornyik: csaba.hornyik@hutton.ac.uk

# Supplementary Figures and Tables

## Supplementary Figures


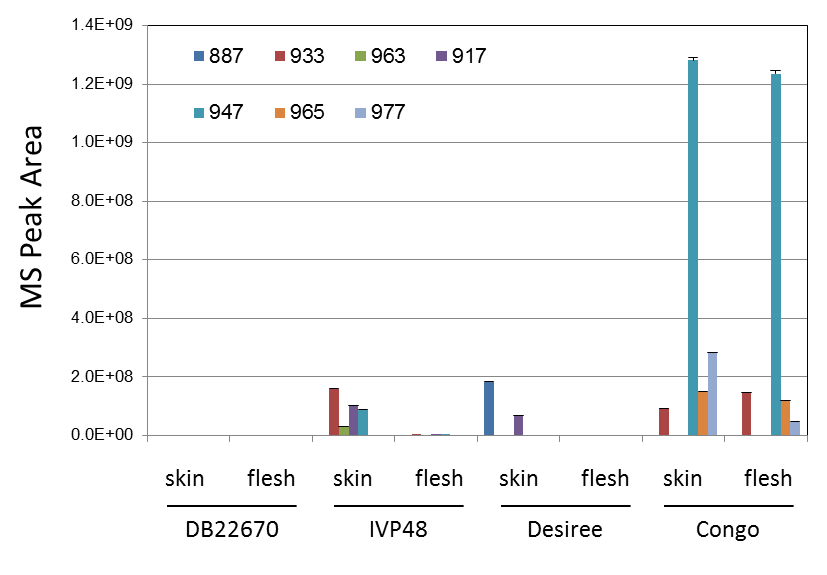


**Supplementary Figure 1.** **Anthocyanin composition in different potato tissues.**

The abundance of each anthocyanin is expressed as average of MS peak areas of duplicate extractions ± SD. The anthocyanins were identified by their *m/z* values as noted below.

The anthocyanins were identified as *m/z* 887 = Pelargonidin 3-*p*-coumaroylrutinoside-5-glucoside; *m/z* 933 = Petunidin 3-*p*-coumaroylrutinoside-5-glucoside; *m/z* 963 = Malvidin-3-caffeoylrutinoside-5-glucoside; *m/z* 917 = Peonidin *3*-*p-*coumaroylrutinoside*-5*-glucoside; *m/z* 947 = Malvidin-3-*p*-coumaroylrutinoside-5-glucoside; *m/z* 965 = unknown possibly Malvidin-3-caffeoylrutinoside-5-glucoside & *m/z* 977 = Malvidin-3-feruloylrutinoside-5-glucoside. All MS data including the MS^2^ fragmentations fits with data from Oertel et al (2017). MS properties of main anthocyanin peaks are in Supplementary Table 4.

>AT3G25795 (TAS4)

TATATAAGTTCTTCCAAAGGTTTCTTTTAATAAGAAAAATAAAAATATGATGCCTTTCATTCATATATCGAGAAAGAGAGCGAGACAGAAACAAATGTTGAAAGAATCGGATCGTATTAGCTTGGTAAACACTACCCTTTTAGTTGATTACTATTTTTTTAAAGTTTATGCATGTTAACGTTTTAACATTCTTTTTTTCTTGTTAAAGCTTACCTTTTCTCTTTTGCTGGAATAATTATTTTATCCATATTTTGGTTTAGGTTATTTTATACACAAAGAATATTTGACCTATTTTTTATTTTTATTTTTATTTTTAACATTAATTTTACTCTTTTTTTTTTGGACAAAAAATTCACTCTATTTTATAATTTGTTTTTCACTTCATTATCTTTCTTCTTCTTTTTTTGATAAATCAGTTCATTATCTTTAACGTCCGCAATACGTAAAACTCGAATTTGGGTTCGTGCTGATGTTTCGCACCTTCAGATTATATTGATTAACTTACTATCACTGGTTCTGGTTTATTTAGGTTTCACTAACAATAAAATATAATGACTAATTTTGCATGCTCATATATACAGTAAAGAGATTTTGTTTAGTAGTAAGAGTTAGTAATTTTGCATGCTCATATATACAAAAAAAAAAAAACCGCCGGTCACTTAATTAACAACACTTGTATAAATGAGGTAAGATTGAAAAATGGGAAGGATATTAACAACAATTACGTAGAGTTAATTAACTTTACGTCAGTCCATGAGATATTAGTTTTATAACTAACACAAACTTTTTTAAGTCACTCAAACACTGACGTGAACCTTATACATATAAACCTTTTTAAGTTTCTTTTTCTTTTGCAGGTTACCAATCACTCTCCATGGAATACTCATTTGAGCAAGATGTTGGCATGAAATTGCCGTGGTGAAGGACGAGCTGACTCTATATCGATGGTGCCTCGACCTCGATCCTTCACCTATTTATTGAAAGCATAGTTTGAATCTCATTTTCAACTATGTATAAATTAATATAAACAATGCAGGTCTAGAAAATAAGCTCTTTTTTTTTCATTACCATTGACTGCCTAAAATAGTGATGTATAACTACTTTTTTACATGTTACTAGTTATGACTTTAAAATAATATACATTATTCTTTTCAA

>SYL_TAS4

TCACTAGGTCTTATTTCCTATTAGTATATAATACTTAGTAAACAATAGGTTATGGCACATGAAAAAGGGAAAAAAAAGTGAGACTTTTACACATGGTGGTTGAGACATAGTTAACCTTAGAAAGGTGGATGTCATGCTTATAAACTTATAATCCTTCAACCACCAAAAAGCTACATTTTTTTATTTTTCCCCCCTATAAATAAGAGCTTCACTTGAATACATTTTCCCATAAAAATCTAACAGTCTCATCTTATACACTTCTAAAAAAATAATAAAATTAGCGATAGCTAAACAACAAAATCTGTTACTAATCCTATTTAGTGACGGATTAGCAACAATTTTCAATATTTTTATTTAGTTACCAGCAATTCAACGACGAAGTTCGTACTATATATATATATACCATTATGTCTCTTCTCAATAATAATATCGTGTCTCTTTTCTTGAGTACCAATAAATCAGATGGAGTAAGAAGAAAAAGAAGGAAAAAACCCTATCGAAGTTTGCTGGATACAATTAGATAAATCATCTGGAACATTCATTTGAGCAAGAAAGTCAGAGTTAGTAAGACGATGAAGGTACGAGGATGATGTTGTTTTAACCAACCTCAACCTCGGACCTTCATCTATAACGTCAATCATTACGCACGACGAAGCGTTCATCACCTCTATTTCACATAATATTTCACTCAAAGAAAATT

>STU_TAS4

TATTGTTTTTTTCAGGTTACAATTTGGTAAATCATTTGGAACATTCATTTGAGCAAGAAAGTTAGAGTTAGTACGACGATGAAGGTACGAGGATGATGTTGTTTTAGCTAACCTCAACCTCGGACCTTCATCTATAACGTCAATTATGACGTAGGCGCACACGTGGAACGTTCATCACCACTATTTCACATAATATTTCACTCAAGAAAATT


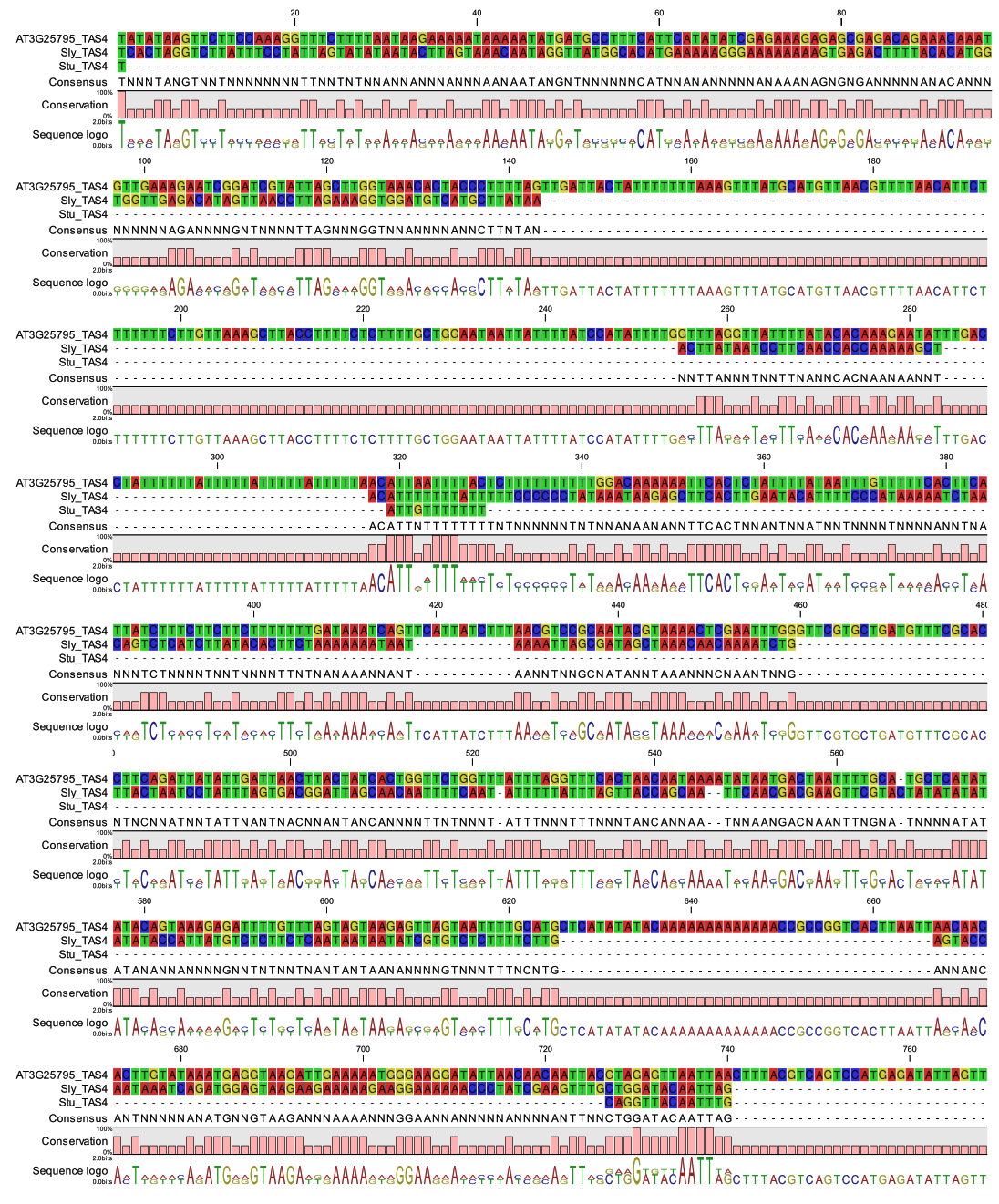


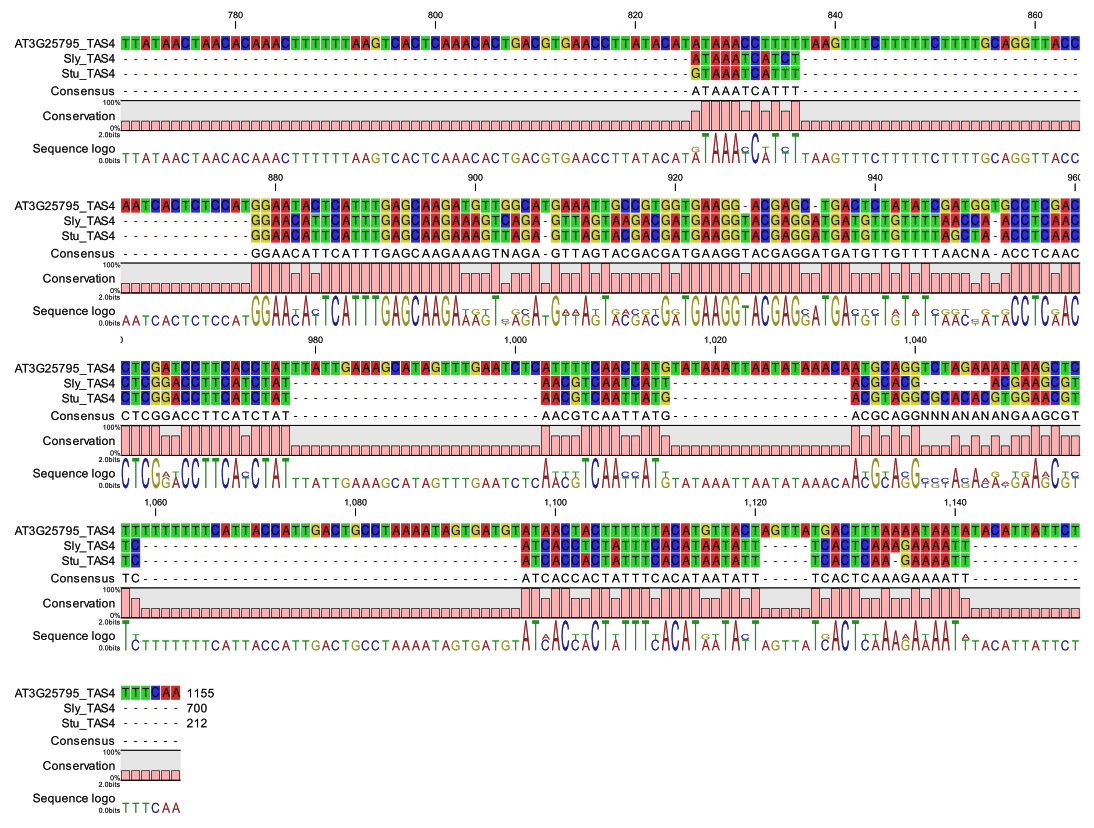


**Supplementary Figure 2.** **The sequences of Arabidopsis (AT3G25795 [TAS4]), tomato (SYL_TAS4) and potato (STU_TAS4) *TAS4* genes.** DNA sequences were used to make the alignment; miR828 binding site and conserved tasiRNA are shown on **Figure 3**.


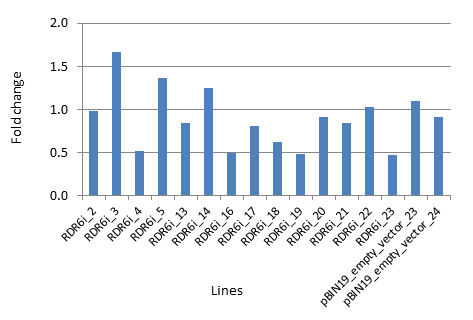


**Supplementary Figure 3.** **Relative transcript levels of RDR6i lines and control plants** (transformed with empty vector, pBIN19_empty_vector). These lines were used to investigate the expression levels of endogenous RDR6 by RT-qPCR.


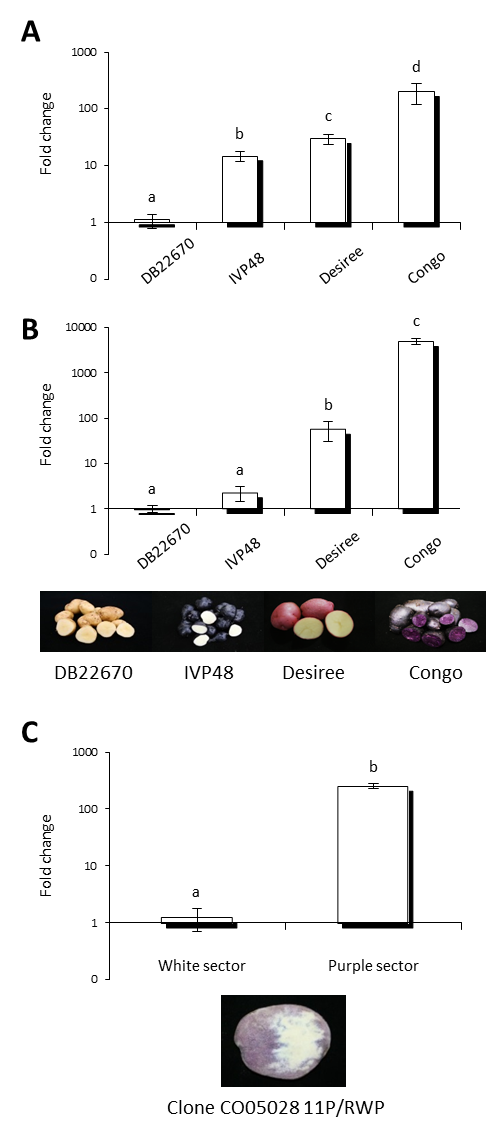


**Supplementary Figure 4.** **Relative transcript levels of *TAS4* RNA.** Skin samples (**A**) and flesh tissues (**B**) of the cultivars were used to investigate the expression levels of *TAS4* RNA by RT-qPCR. Purple and white sectors (**C**) of clone CO0502811P/RWP was investigated for *TAS4* expression. Relative quantification was made to DB22670 and white sector samples; the letters on the bars indicate significantly different expression levels of *TAS4* using Student’s T-test (p<0.05); different letters mean significant differences between the samples.

**B**


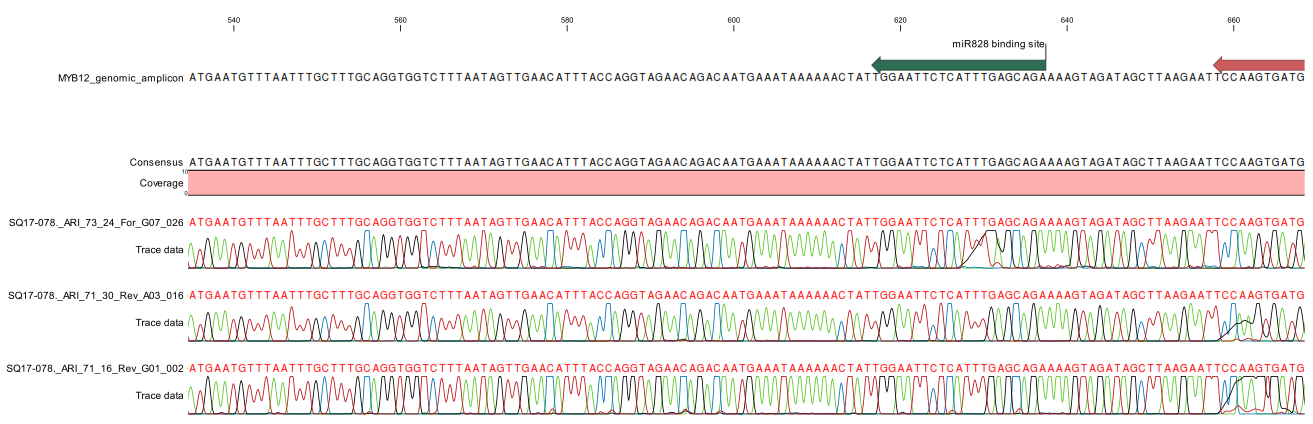

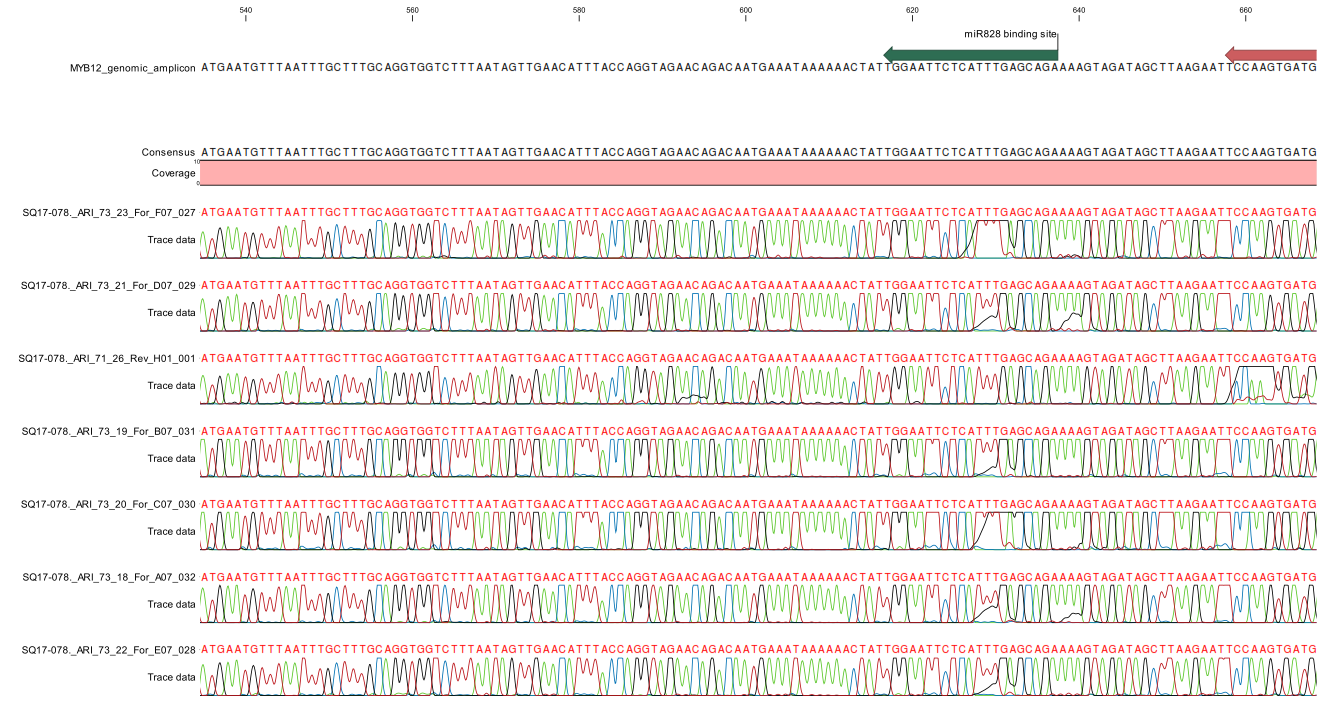

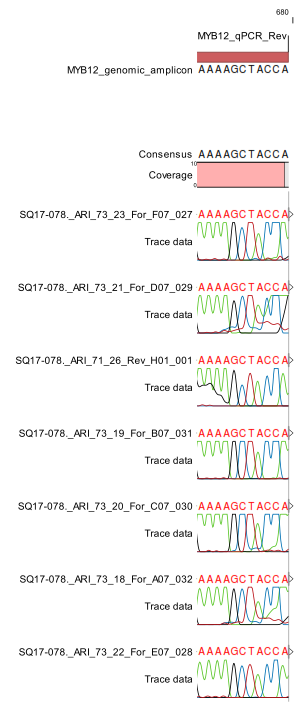

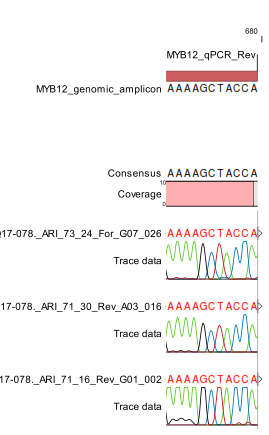


**A**


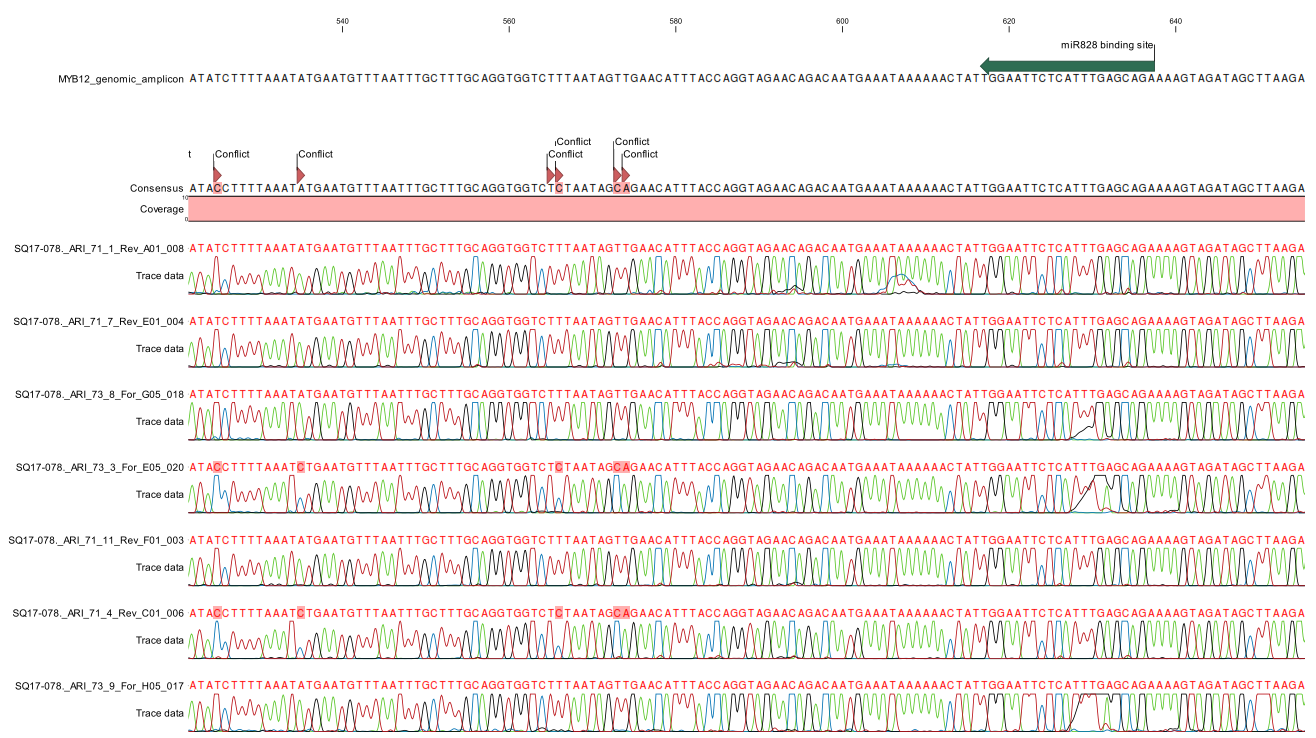

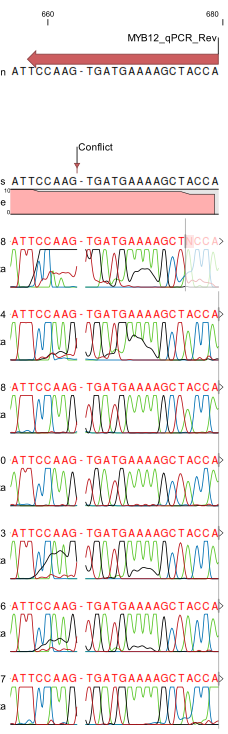

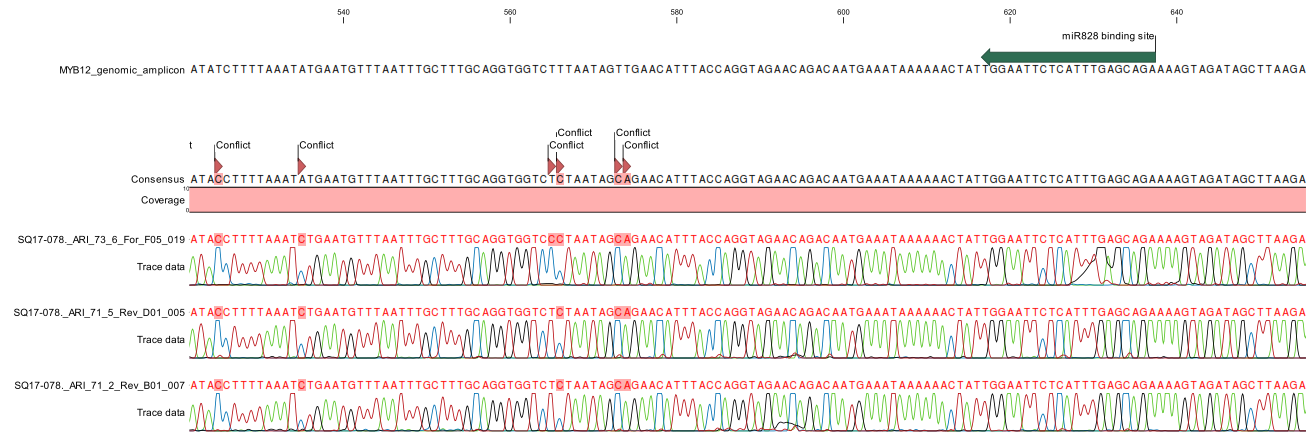

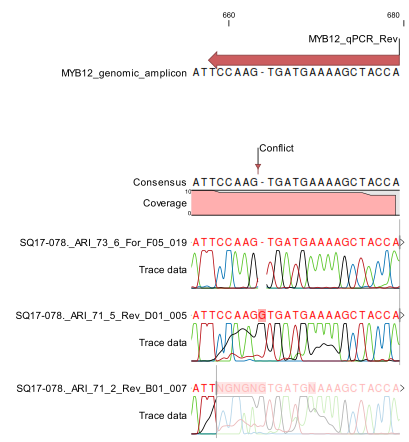

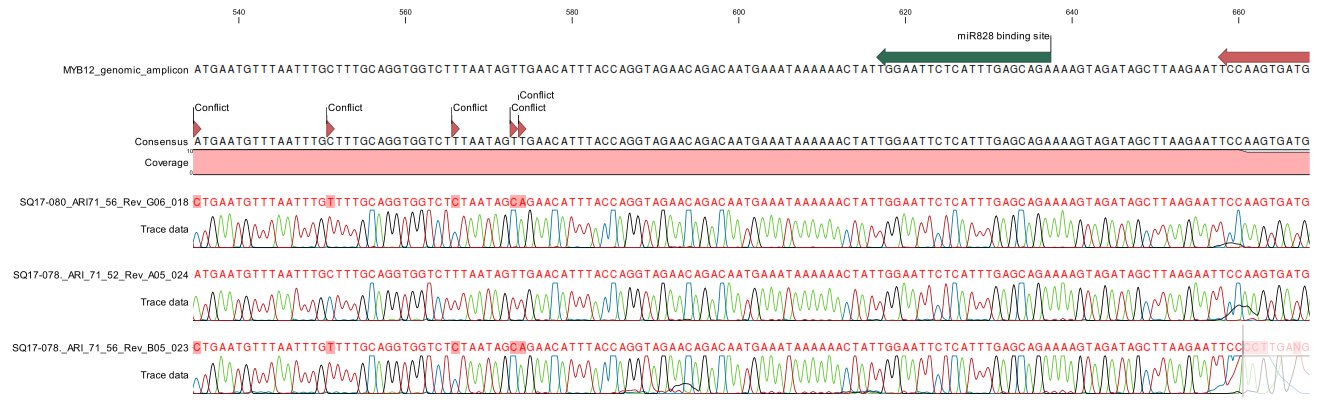

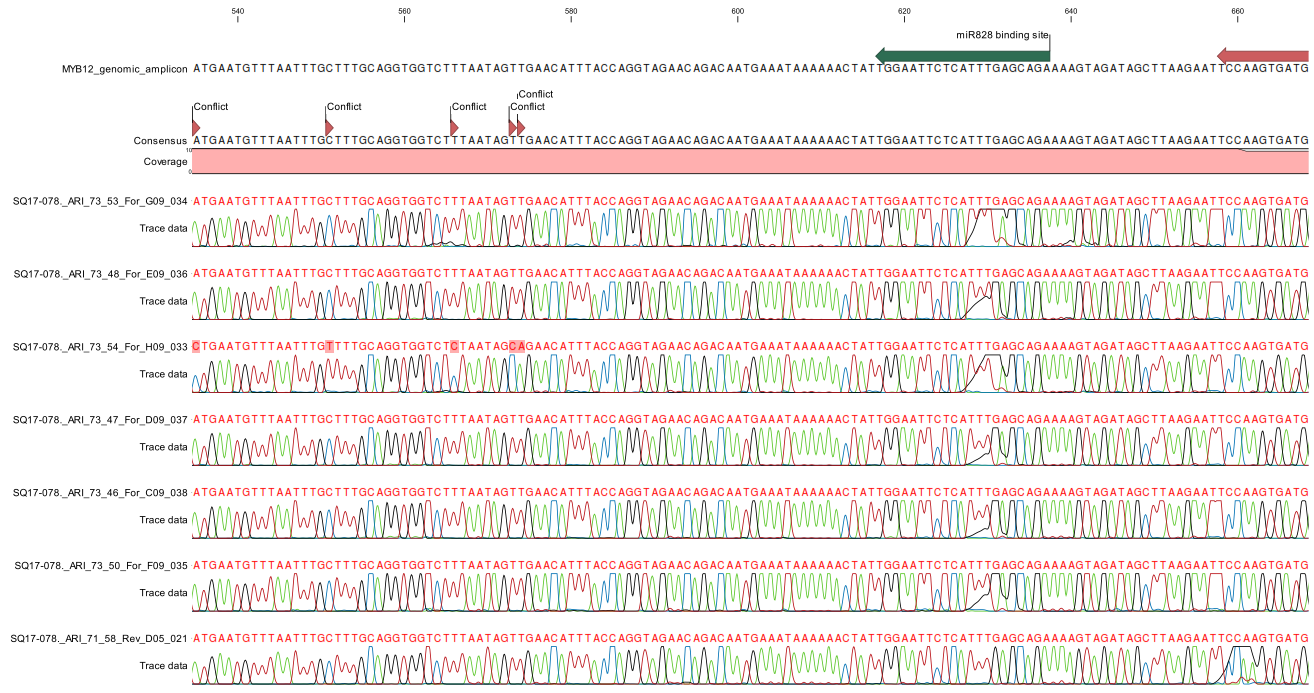

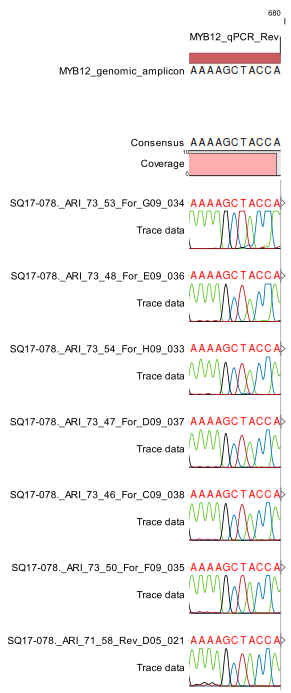

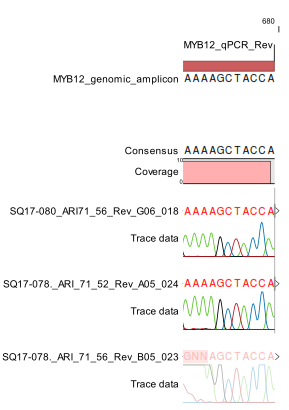

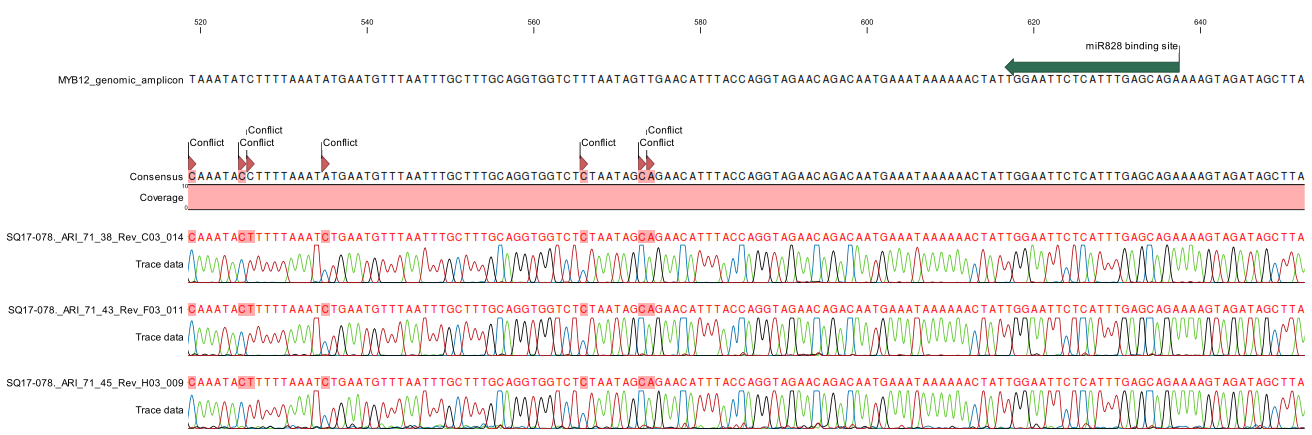

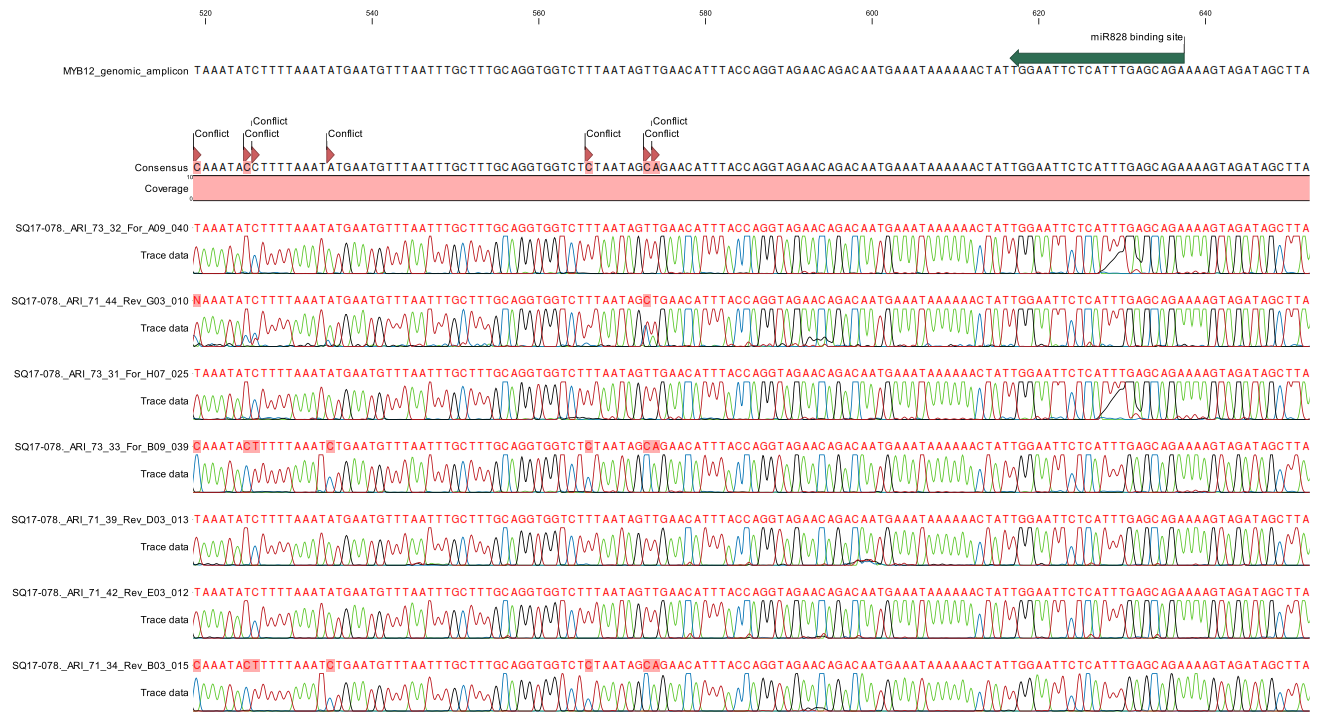

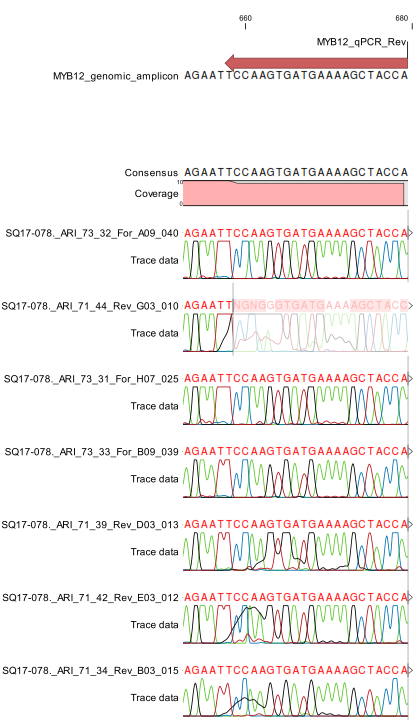

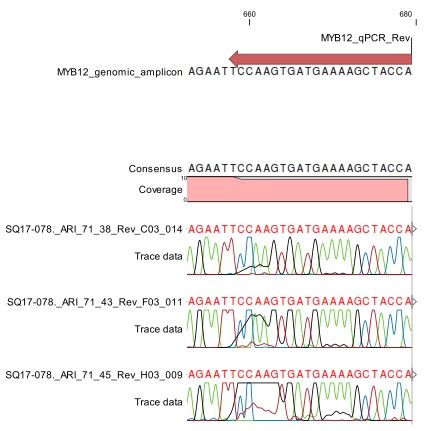


**D**

**C**

**
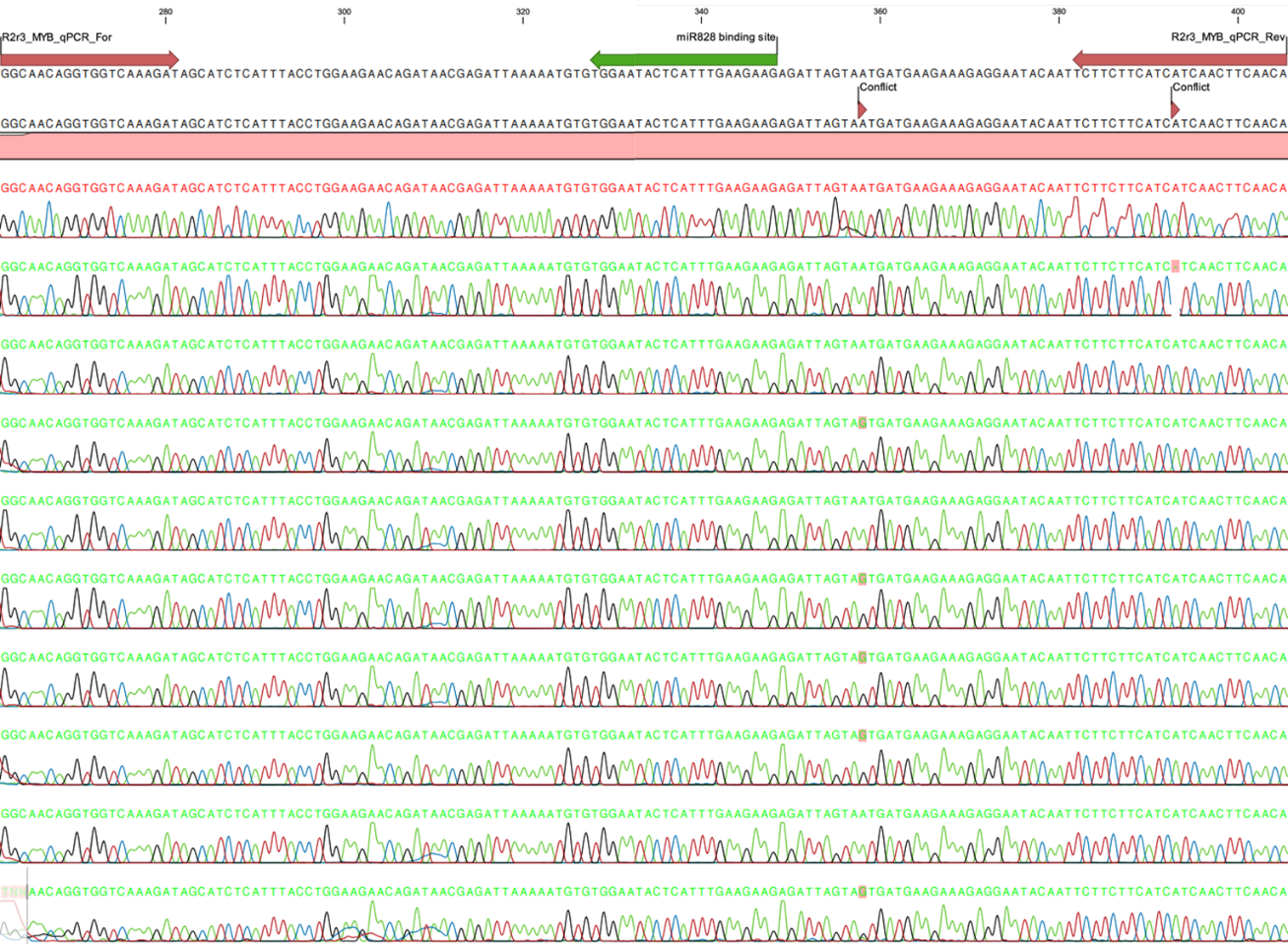

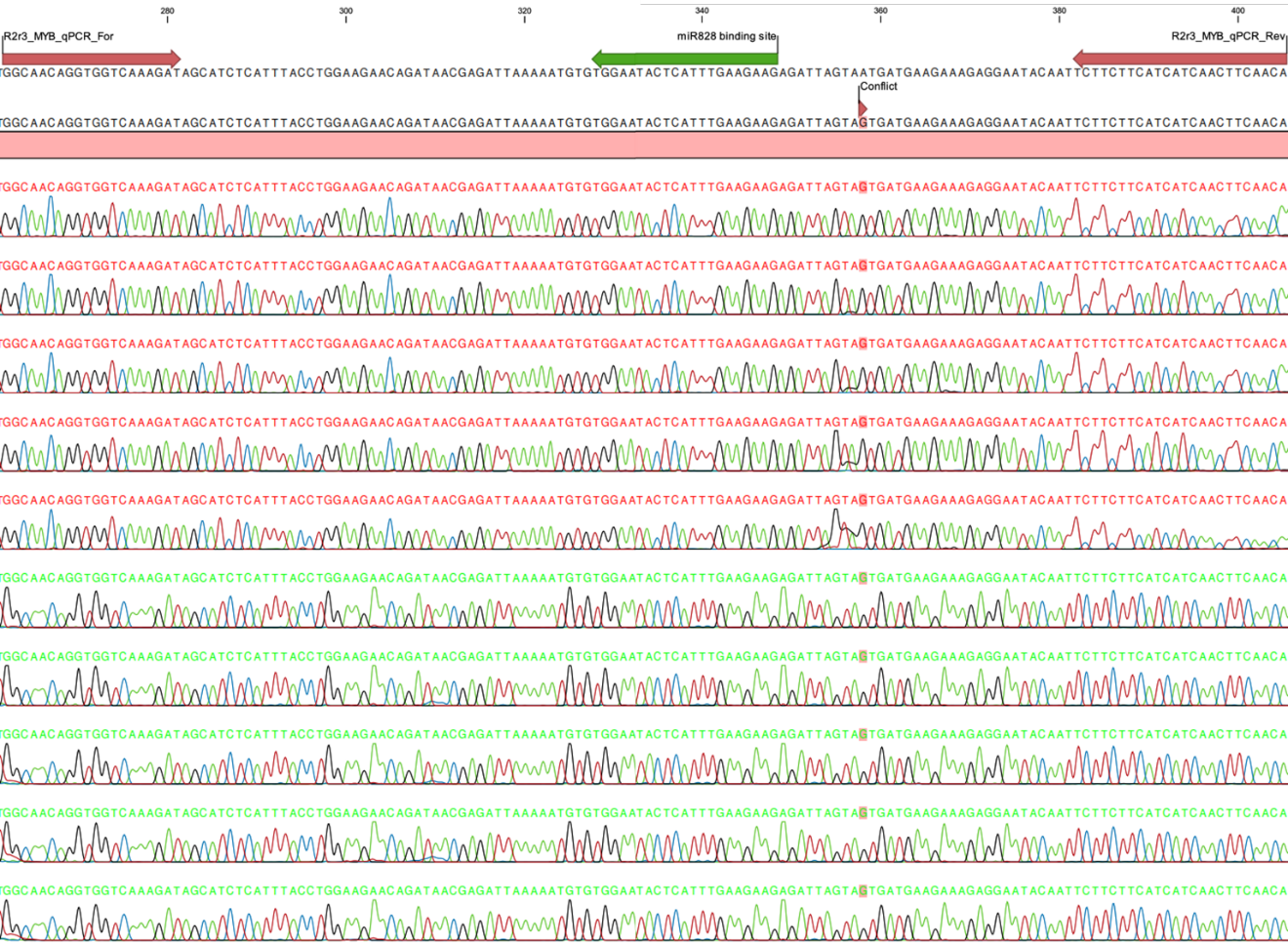
**

**E**

**F**

**
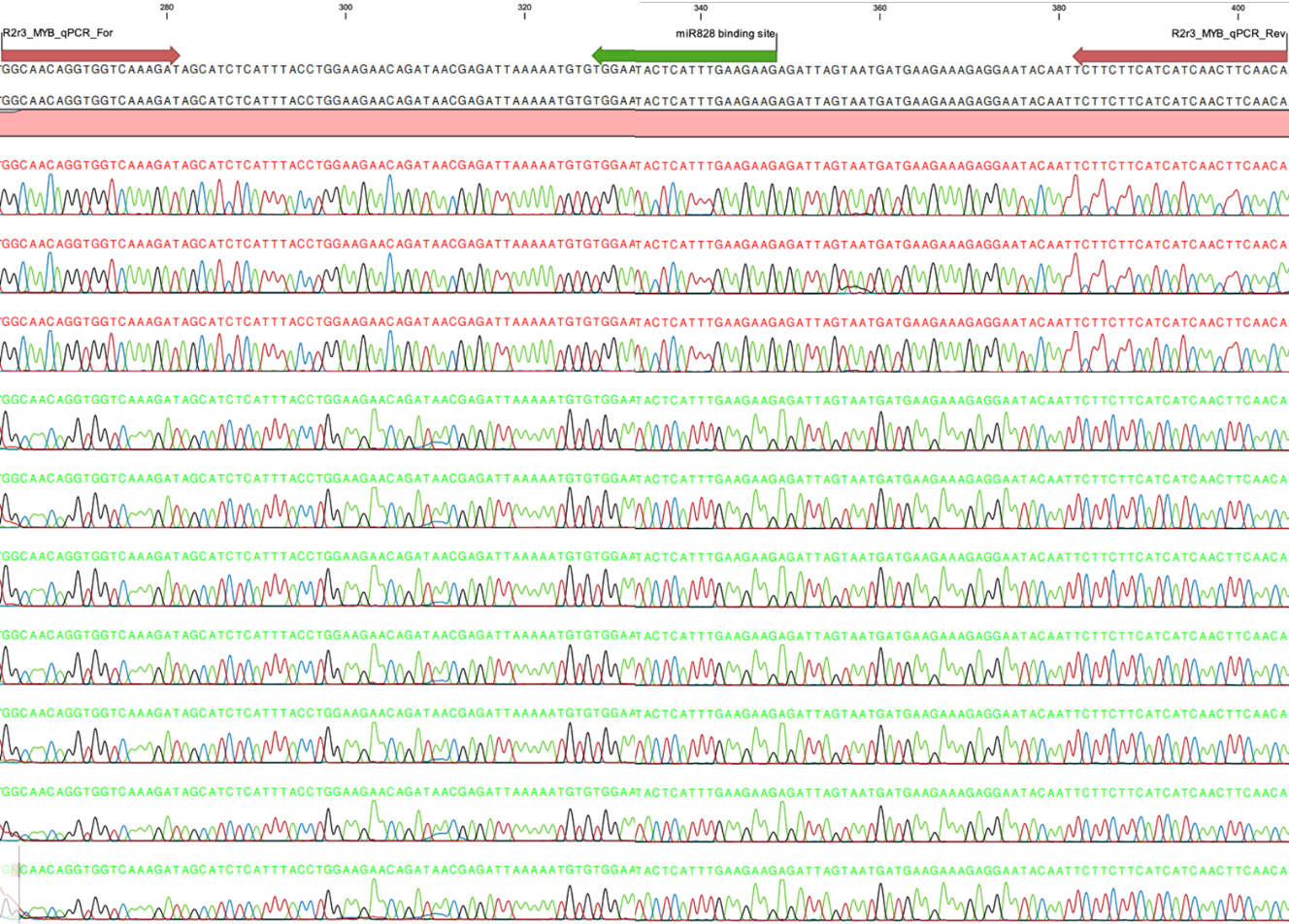

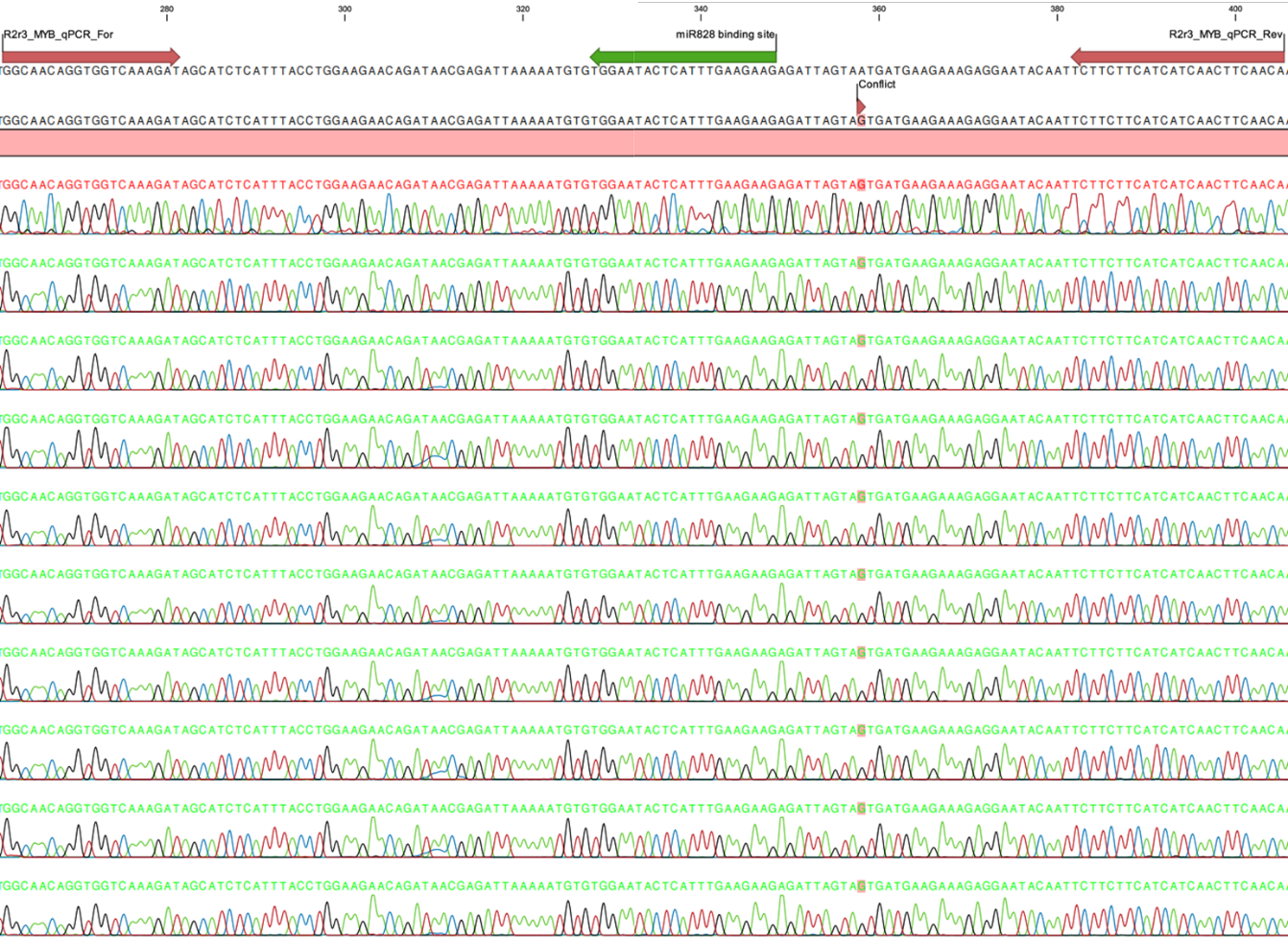
**

**G**

**H**

**
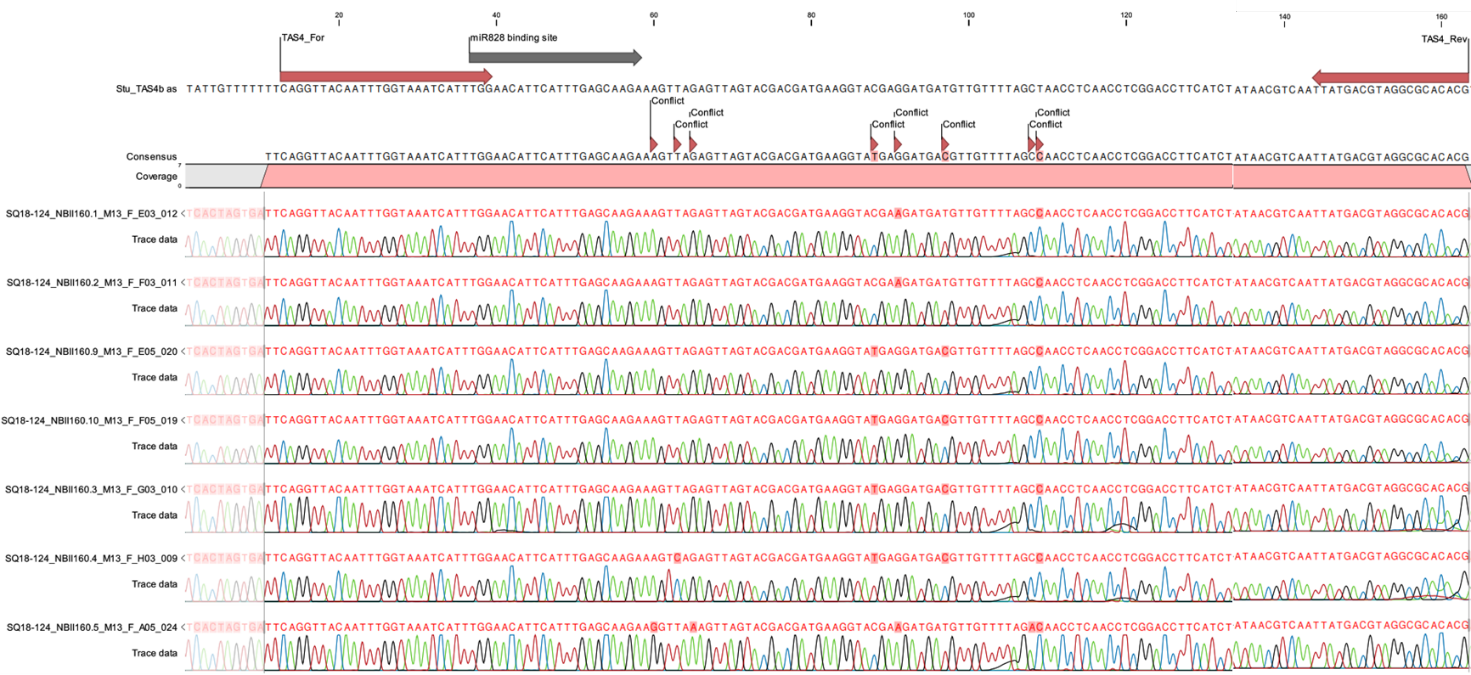

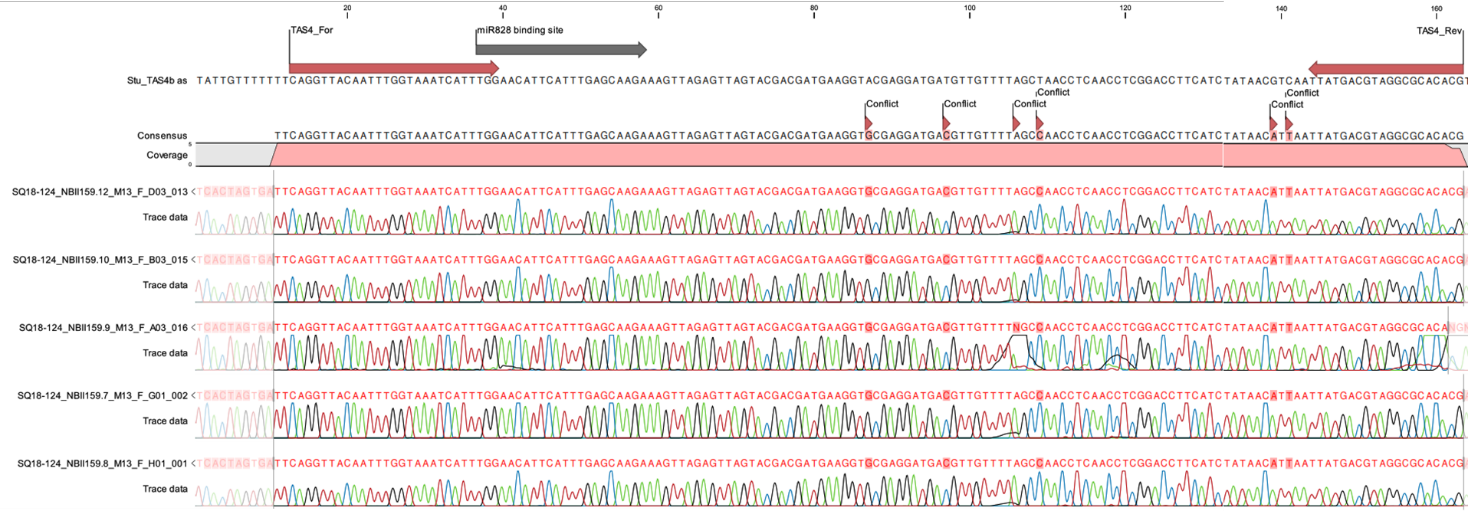

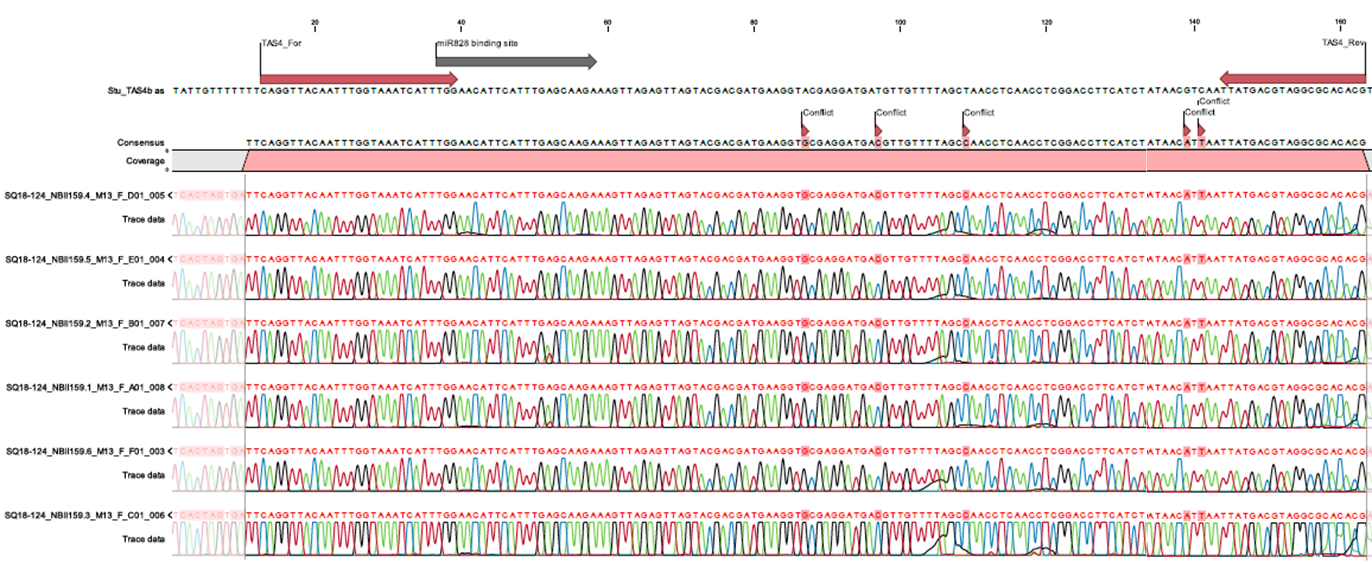
**

**K**

**J**

**I**

**
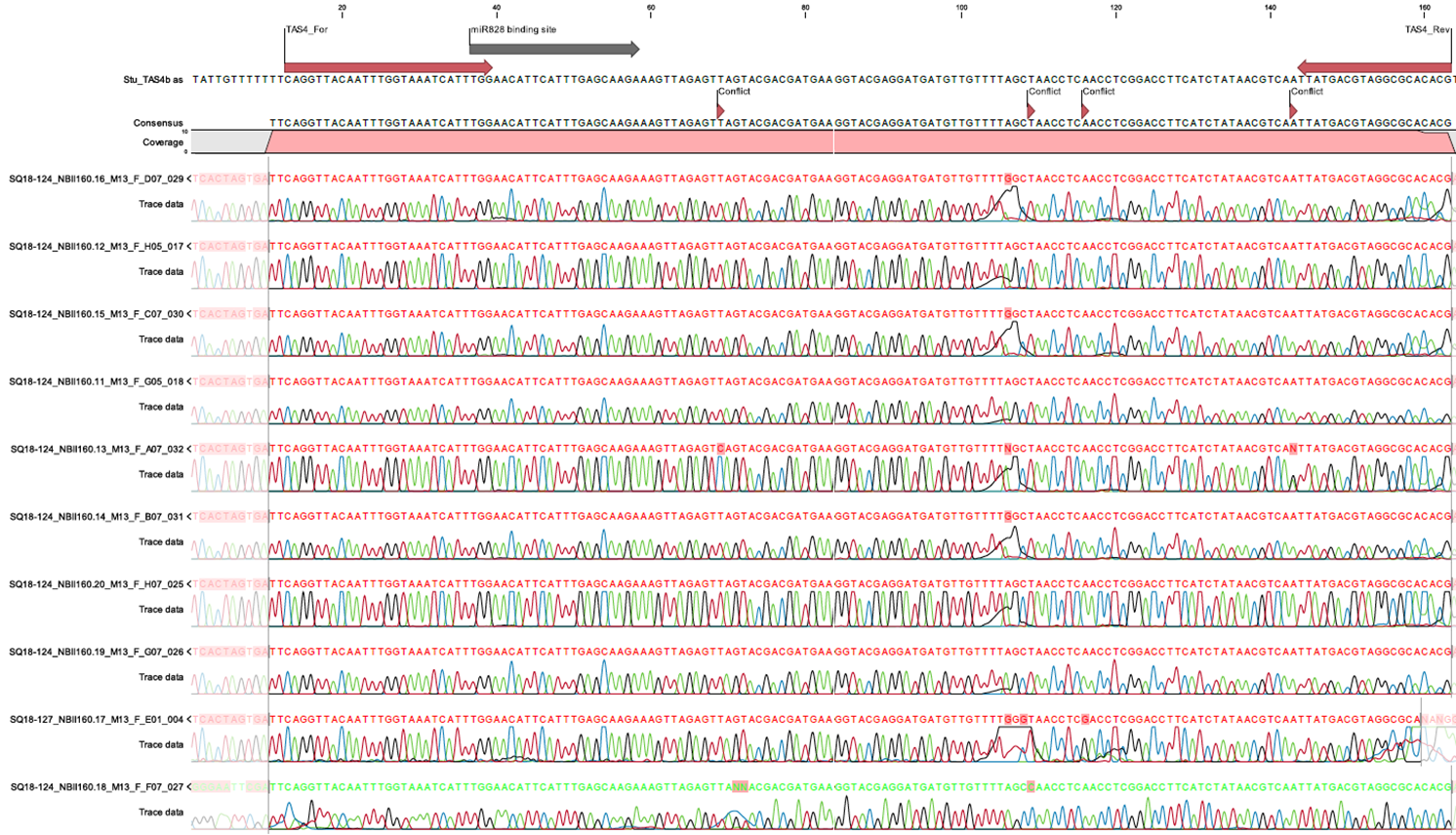
**

**L**

**
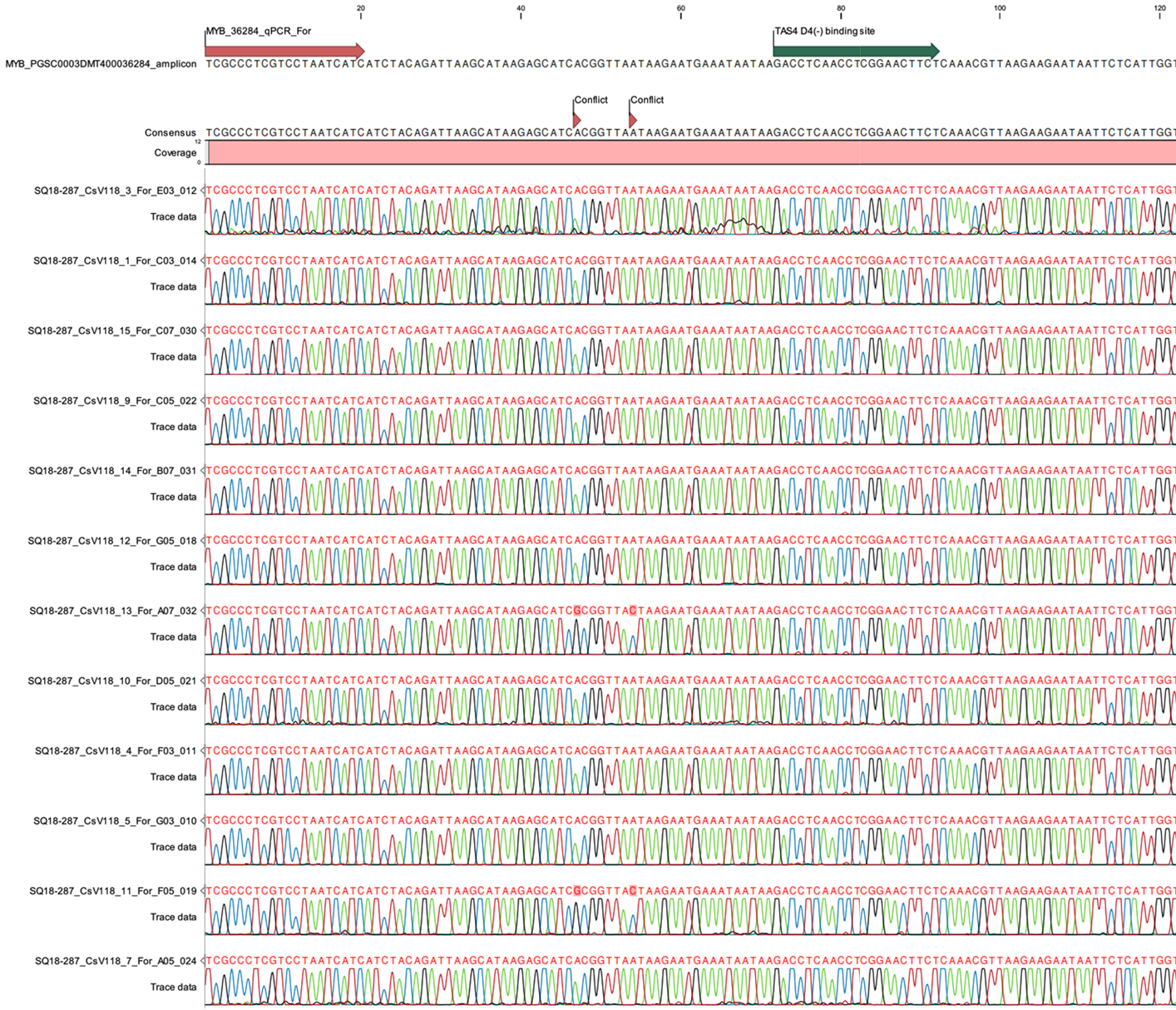
**

**M**

**
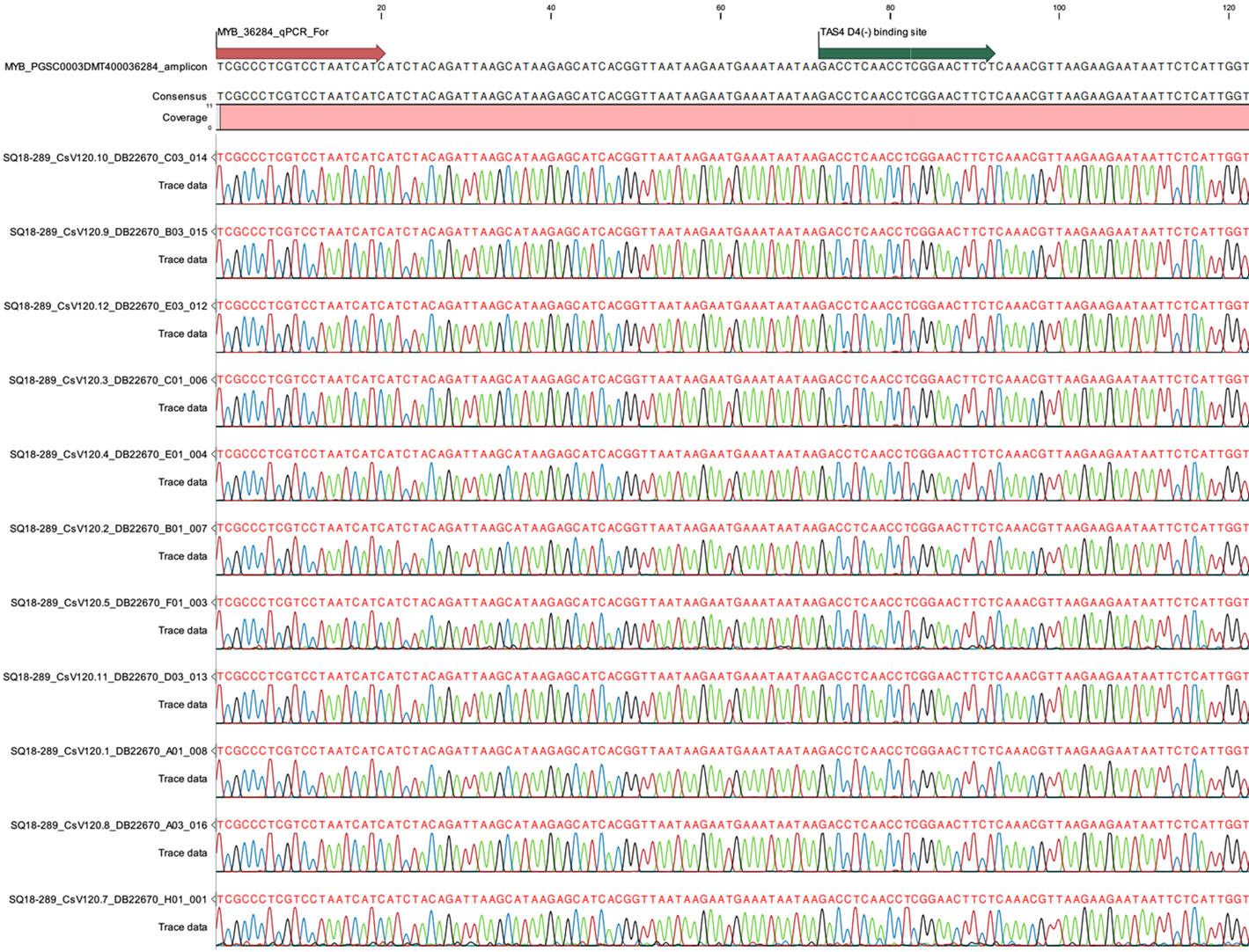
**

**N**


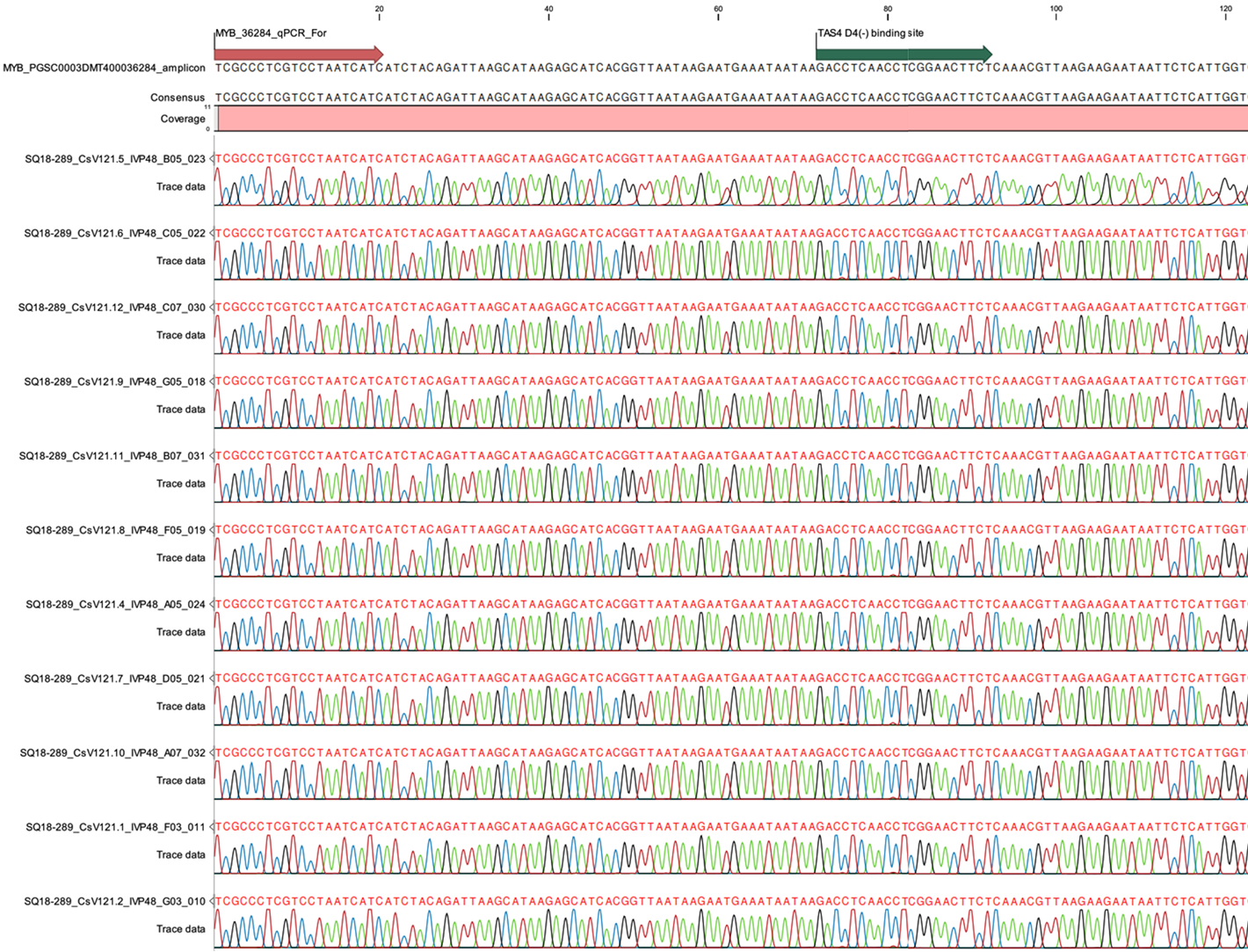


**O**


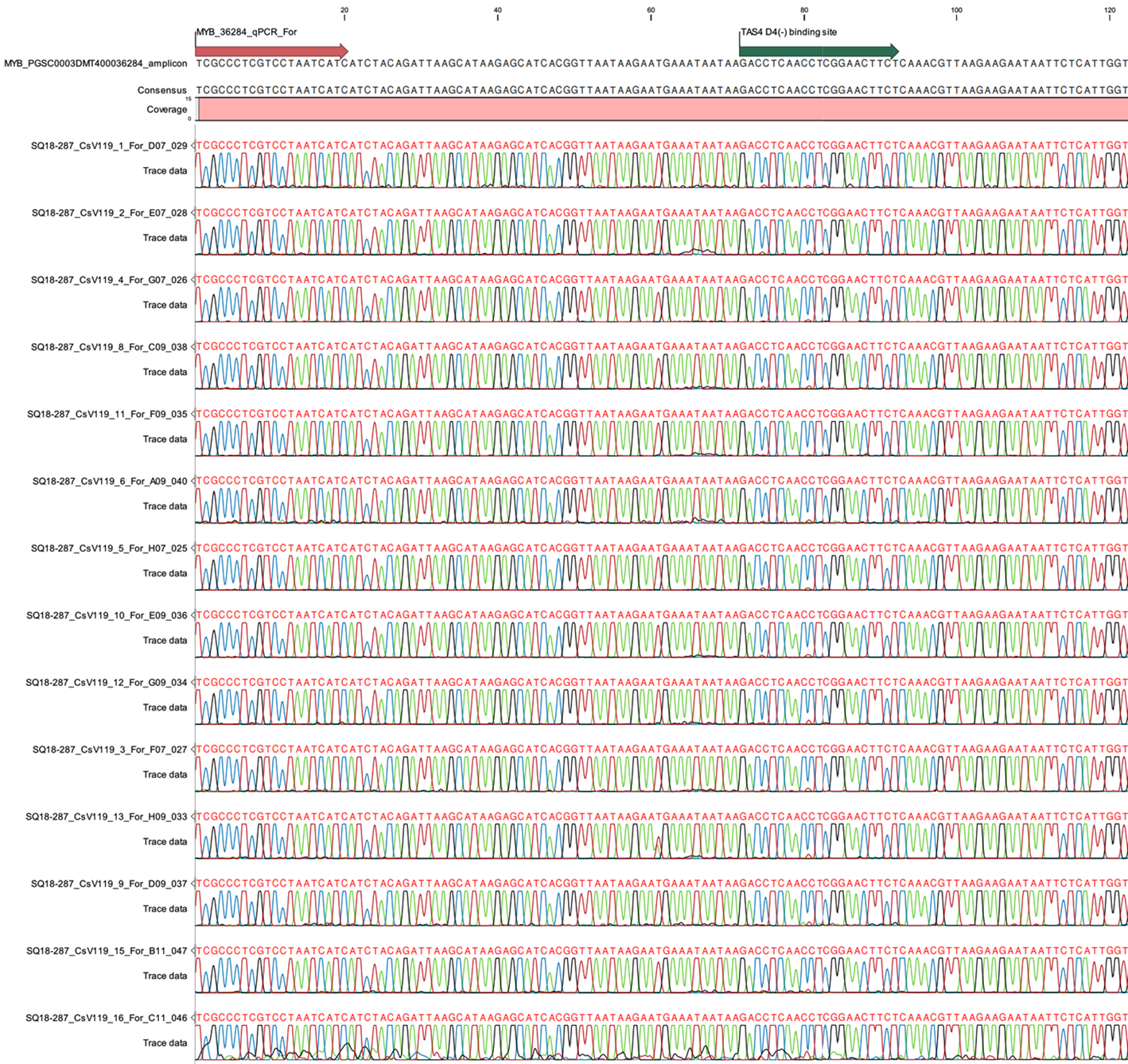


**P**

**Supplementary Figure 5.** **Investigation of small RNA target sites in different cultivars.** RT-qPCR primers were used to amplify the miR828 and TAS4 D4(-) target site regions of *MYB12* gene in Desiree (**A**), DB22670 (**B**), IVP48 (**C**) and Congo (**D**); *R2R3MYB* gene in Desiree (**E**), DB22670 (**F**), IVP48 (**G**) and Congo (**H**); *TAS4* gene in Desiree (**I**), DB22670 (**J**), IVP48 (**K**) and Congo (**L**); *MYB-36284* gene in Desiree (**M**), DB22670 (**N**), IVP48 (**O**) and Congo (**P**).

PCR amplified products were cloned into pGEM-T Easy vector system and individual clones were sequenced with M13 forward primers by Sanger sequencing. Sequencing results were visualized and aligned to the DM sequence of the target genes using CLC main workbench software. Small RNA target sites and PCR primers are indicated on the top of the alignments. Conflict signs show the presence of SNPs in the amplified regions, but no SNPs were detected in the small RNA target sites.

## Supplementary Tables

| Contig | PGSC0003DMB000000382 |
| --- | --- |
| Strand | - |
| miR ID | S_PotatoMir1030981382_x1329 |
| Precursor ID | PGSC0003DMB000000382_5473 |
| miR Location | 252455..252476 |
| Precursor Location | 252408..252476 |
| miR Sequence | TCTTGCTCAAATGAGTATTCCA |
| miR Family | miR828 |
| Precursor Sequence | TCTTGCTCAAATGAGTATTCCAACAAATGTCTAATTGACATACTTTGAGATACTCATTTGGGCAAGAAG |
| Star sequence | AGATACTCATTTGGGCAAGAAG |
| miR reads | 1329 |
| star reads | 359 |

**Supplementary Table 1.** **miR828 prediction.** The table shows the details of predicted miR828; the super-scaffold location (Contig); the direction of chromosome strand where the miRNA maps (Strand); read and precursor identification number according to the analysis (miR ID, Precursor ID); the location of miRNA and precursor RNA on the super-scaffold (miR Location, Precursor Location); the mature sequence of the miRNA (miR Sequence); the family where the miRNA belongs to (miR Family); the precursor sequence (Precursor Sequence); the star sequence (Star sequence) and read number for miRNAs and star sequences (miR reads, star reads). During the analysis DNA equivalent of RNA sequence was used.

**Supplementary Table 2.** **Target prediction of miR828 and TAS4 D4(-).** The tables contain the small RNAs which were used for target prediction (miRNA Acc.), the homologue of miRNA (miRNA Annotation) and the transcript number of targeted potato RNAs (Target Acc.) with the predicted gene functions (Target annotation). Expectation score (Expectation) and unpaired energy (UPE) with a low number suggest highly likely target for the small RNA. The start and end nucleotides of aligned miRNA and target RNA are indicated with the corresponding sequences (miRNA/Target start and end, miRNA/Target aligned fragment). The inhibition type (Inhibition) and the number of target sites (multiplicity) are shown. Green highlight shows the genes investigated in this study.

| Gene | PGSC ID | Purpose | Forward | Reverse | UPL porbe number |
| --- | --- | --- | --- | --- | --- |
| StEF1a | PGSC0003DMT400059830 | RT-qPCR | CTTGACGCTCTTGACCAGATT | GAAGACGGAGGGGTTTGTCT | #117 |
| MYB12 | PGSC0003DMT400018841 | RT-qPCR | CAGGTGGTCTTTAATAGTT | TGGTAGCTTTTCATCACTTGGA |  |
| R2r3_MYB | PGSC0003DMT400029235 | RT-qPCR | GGCAACAGGTGGTCAAAGAT | TGTTGAAGTTGATGATGAAGAAGA |  |
| MYB_36284 | PGSC0003DMT400036284 | RT-qPCR | TCGCCCTCGTCCTAATCATC | TTTCCTTGCAACGTTTGTCG |  |
| TAS4 |  | RT-qPCR | CAGGTTACAATTTGGTAAATCATTTGG | CGTGTGCGCCTACGTCATAA |  |
| R2r3_MYB | PGSC0003DMT400029235 | outer 5' RACE primer |  | GGCATTCAAATTCATCTACAAGCTGCTG |  |
| R2r3_MYB | PGSC0003DMT400029235 | inner 5' RACE primer |  | TCATTAGCCTTGGAAGAAGTGGGAGA |  |
| R2r3_MYB | PGSC0003DMT400029235 | control primer 5' RACE | TCCCACTTCTTCCAAGGCTA | TCAACCACTTTTGGCATTCA |  |
| TAS4 |  | outer 5' RACE primer |  | CGTGTGCGCCTACGTCATAA |  |
| TAS4 |  | inner 5' RACE primer |  | GGTCCGAGGTTGAGGTTAGC |  |
| TAS4 |  | control primer 5' RACE | CGACGATGAAGGTACGAGGA | CGTGTGCGCCTACGTCATAA |  |
| MYB_36284 | PGSC0003DMT400036284 | outer 5' RACE primer |  | TGTTGCATGGAATTGCCTTCACCATT |  |
|  | PGSC0003DMT400036284 | inner 5' RACE primer |  | CGACGATGTATTTTCTCCTGTTGGCTTC |  |
|  | PGSC0003DMT400036284 | control primer 5' RACE | TGGAGTTCAATGGTGGACAA | CATGGAATTGCCTTCACCAT |  |
| stu_miR828 |  | small RNA blot, probe sequence |  | TGGAATACTCATTTGAGCAAGA |  |
| TAS4_D4 |  | small RNA blot, probe sequence | AACCTCAACCTCGGACCTTCA |  |  |
| U6 |  | small RNA blot, probe sequence |  | GCTAATCTTCTCTGTATCGTTCC |  |
| RDR6_UPL | PGSC0003DMT400060673 | RT-qPCR | TGAAAATCTCATTCCACCCAGT | TTCTGCAGGTGCATAGTCCA | #42 |
| StRDR6_RNAi | PGSC0003DMT400060673 | Gateway cloning | GGGGACAAGTTTGTACAAAAAAGCAGGCTATAATGTTGAGTGCAGGGT | GGGGACCACTTTGTACAAGAAAGCTGGGTCTCCAGAATCCTCACATCC |  |

**Supplementary Table 3.** **Oligonucleotide sequences used in this study.**

| *m/z* [M+H]^+^ | **887** | **933** | **963** | **917** | **947** | **965** | **977** |
| --- | --- | --- | --- | --- | --- | --- | --- |
| MS^2^ | 725, 433, 271 | 771, 479, 317 | 801, 493, 331 | 755, 463, 301 | 785, 493, 331 | No signals | 815, 493, 331 |

**Supplementary Table 4.** **MS properties of main anthocyanin peaks.** Major fragment ions are underlined.
